# Impact of selenium supplementation on fish antiviral responses: a whole transcriptomic analysis in rainbow trout (*Oncorhynchus mykiss*) fed supranutritional levels of Sel-Plex®

**DOI:** 10.1186/s12864-016-2418-7

**Published:** 2016-02-16

**Authors:** D. Pacitti, M. M. Lawan, J. Feldmann, J. Sweetman, T. Wang, S. A. M. Martin, C. J. Secombes

**Affiliations:** Scottish Fish Immunology Research Centre, Institute of Biological and Environmental Sciences, University of Aberdeen, Aberdeen, AB24 2TZ UK; Trace Element Speciation Laboratory, Department of Chemistry, University of Aberdeen, Aberdeen, AB24 3UE UK; Alltech Biosciences Centre, Sarney, Summerhill Rd, Dunboyne, County Meath Ireland; Present address: Department of Cellular and Physiological Sciences, University of British Columbia, Vancouver, BC Canada

**Keywords:** Selenium supplementation, Sel-Plex, Poly(I:C), Immune response, Antiviral response, Microarray, Fish

## Abstract

**Background:**

Selenium (Se) is required for the synthesis of proteins (selenoproteins) with essential biological functions. Selenoproteins have a crucial role in the maintenance of cellular redox homeostasis in nearly all tissues, and are also involved in thyroid hormone metabolism, inflammation and immunity. Several immune processes rely on Se status and can be compromised if this element is present below the required level. Previous work has supported the notion that when Se is delivered at levels above those deemed to be the minimal required but below toxic concentrations it can have a boosting effect on the organism’s immune response. Based on this concept Se-enriched supplements may represent a valuable resource for functional feeds in animal farming, including aquaculture.

**Results:**

In this study we tested the effects of Se supplemented as Sel-Plex during an immune challenge induced by polyinosinic:polycytidylic acid (poly(I:C)), a pathogen-associated molecular pattern (PAMP) that mimics viral infection. Trout were fed two diets enriched with 1 or 4 mg Se Kg^−1^ of feed (dry weight) by Sel-Plex addition and a commercial formulation as control. The whole trout transcriptomic response was investigated by microarray and gene ontology analysis, the latter carried out to highlight the biological processes that were influenced by Sel-Plex supplementation in the head kidney (HK) and liver, the main immune and metabolic organs in fish. Overall, Sel-Plex enrichement up to 4 mg Se Kg^−1^ induced an important response in the trout HK, eliciting an up-regulation of several genes involved in pathways connected with hematopoiesis and immunity. In contrast, a more constrained response was seen in the liver, with lipid metabolism being the main pathway altered by Se supplementation. Upon stimulation with poly(I:C), supplementation of 4 mg Se Kg^−1^ increased the expression of principal mediators of the antiviral defences, especially IFN-γ, and down-stream molecules involved in the cell-mediated immune response.

**Conclusions:**

Supplementation of diets with 4 mg Se Kg^−1^ using Sel-Plex remarkably improved the fish response to viral PAMP stimulation. Sel-Plex, being a highly bioavailable supplement of organic Se, might represent a suitable option for supplementation of fish feeds, to achieve the final aim of improving fish fitness and resistance against immune challenges.

**Electronic supplementary material:**

The online version of this article (doi:10.1186/s12864-016-2418-7) contains supplementary material, which is available to authorized users.

## Background

Selenium (Se) is an essential element in human and animal nutrition [1, 2]. Se deficiency is an endemic problem in several parts of the world, that can affect both human and livestock health. A low intake of this element can cause physiological dysfunction and may make the organism more susceptible either to infection or environmental stressors [3, 4]. Se supplementation in farmed animal feed may counteract problems caused by deficiency of this mineral but also ameliorate the physiological response to infection, inflammatory disorders and stress [5]. However, Se supplementation is still controversial, because 1) Se has a narrow range between nutritive requirements and toxicity, 2) nutritional requirements may vary considerably across species and 3) physiological conditions within an organism, as well as environmental factors, can influence the dietary needs for this mineral.

Previous studies suggest that fish require between 0.1 and 0.5 mg Kg^−1^ Se (dry mass): according to these data, fish can regulate Se bioaccumulation through excretion up to 3 mg Kg^ˉ1^ (dry mass) but beyond this level Se starts to exert detrimental effects [6–8]. In accordance with these studies, the European Union has legislated that if additional Se is added to feed, the total level must not exceed 0.5 mg Kg^−1^ (dry mass) (Commission Regulation EU No 432/2012). However these studies need to be updated as they are primarly based on feeding trials using inorganic Se (mainly sodium selenite, Na_2_SeO_3_) as supplement. Recent evidence suggests that organic Se is more bioavailable and tolerated at higher concentrations than inorganic Se [9], where selenomethionine (SeMet) is currently the most preferred alternative for supplementation.

An increasing number of feed suppliers for the aquaculture industry are beginning to substitute fish meal and fish oil with plant sourced materials, which may further lower Se (and other oligonutrients) availability in fish diets. It is important that the diet formulations meet the mineral nutritional requirements of fish as this is crucial to ensure optimal growth and production efficiency in fish farming. In line with this reasoning, a recent study carried out in sea bass (*Dicentrarchus labrax*) showed that fish may benefit from organic Se supplementation up to 5 mg Kg^−1^ (dry mass) in the diet during larval development, due to enhanced antioxidant protection during muscle development [10]. Other investigations in trout have shown that farmed fish may require a higher content of Se in their diet, especially when subjected to stress caused by crowded conditions. In trout, it has been reported that the Se requirement can increase up to 4 mg Kg^−1^ (dry mass) when the fish is subjected to stress and a high tolerance can be ensured if it is delivered in an organic form [11, 12].

Se plays an important role within the immune system [5, 13]. Selenoproteins such as glutathione peroxidases (GPxs) and thioredoxin reductases (TrxRs) are responsible for the regulation of cellular redox status during an immune response [14]. Tight control of radical oxygen species (ROS) produced during an immune challenge is crucial to guarantee effective responses to pathogens. ROS are generated in the early stage of immune activation and they function as mediators of intracellular signalling, as well as paracrine messengers for immune cell recruitment [15]. ROS also have a microbial killing function in phagocytic leukocytes, such as macrophages and neutrophils, i.e. via the oxidative burst [16]. However, oxygen radicals at high levels are cytotoxic bioproducts capable of damaging lipids, proteins and nucleic acids [17]. Se incorporated within selenoproteins exerts its antioxidant activity by maintaining the balance between the positive and negative effects of ROS [14]. Other selenoproteins, strictly related to inflammation and immunity, include the endoplasmic reticulum (ER) transmembrane proteins selenoprotein K (SelK) and S (SelS), which are both involved in protection of cells towards ER stress. SelS appears to be involved in retrograde translocation of misfolded proteins from the ER [18], whereas SelK is required for Ca^2+^ flux during the activation of different immune cell types (such as T cells, neutrophils, and macrophages) [19].

Previous studies support the possibility that Se supplementation can have beneficial and boosting effects on an organism’s immune response. In this context, Se-enriched feed additives have become an attractive resource for formulation of functional feeds for animal farming, including aquaculture. However, the use of Se supplementation to improve farmed fish defences towards infections is still controversial, since it is difficult to determine in different species which level can be beneficial before detrimental effects due to Se toxicity occur. From our previous study in rainbow trout [20] it was clear that Se supplemented as Se-yeast (Sel-Plex) is well absorbed up to 8 mg Se Kg^−1^ (dry mass) without causing any evident sign of toxicity. In addition the metabolic response of trout to Sel-Plex shows a clear change in the profile of selenocompound accumulation with increasing concentration of Sel-Plex inclusion: at concentrations ≥ 4 mg Se Kg^−1^ there was evident accumulation of selenocysteine (SeCys) over SeMet, which may account for the higher selenoprotein synthesis (Lawan, pers observation). However, when the transcriptomic response of several selenoproteins was analysed, a higher induction of their mRNA expression was seen primarly when 0.5 and 4 mg Kg^−1^ of Se was added to the diet. For continuity of these investigations, in this study we tested the effects of Sel-Plex supplementation during an immune challenge induced by poly(I:C), a double stranded RNA that mimics a viral infection. The whole trout transcriptomic response was investigated by microarray analysis and gene ontology (GO) analysis, the latter carried out to highlight the biological processes that were most influenced by Sel-Plex supplementation in the head kidney (HK) and liver, the principal immune and metabolic organs in fish, respectively.

## Methods

### Fish maintenance and experimental design

All procedures were carried out under the UK Animals (Scientific Procedures) Act 1986 and Home Office code of Practice guidance, under Home Office project license PPL 60/4013, approved by the ethics committee at the University of Aberdeen.

Rainbow trout (~75 g) were obtained from Mill Trout Farm (Almondbank, Perth, UK) and kept in 1-m-diameter fiberglass tanks supplied with recirculating freshwater at 15 ± 1 °C, containing 50 mg/l of dissolved oxygen, within the aquarium facilities in the School of Biological Sciences, University of Aberdeen. For the feeding trial, 216 fish were distributed in nine tanks; three tanks were assigned to each diet, giving 72 fish per diet group. After an acclimatization period of four weeks, a ten week feeding trial was carried out. Three diets were used: the acclimatization diet as control and two diets enriched with Sel-Plex to give an additional 1 or 4 mg Se Kg^−1^. All the diets were prepared by the Hellenic Centre for Marine Research, Greece (HCMR, www.hcmr.gr) and the composition is given in Table 1 and Additional file 3: Table S1 . All the components were kept constant with the exception of the wheat meal and wheat gluten that were reduced in proportion to the Sel-Plex addition (ie to ensure the oil and protein content were the same between diets). Briefly, the vitamins and Sel-Plex were stirred in a small mixer (Kenwood KM260), whilst the remaining raw materials (excluding the fish oil) were placed in a large mixer (Hobart A200 DT). After mixing for 15 min, the two components were combined together in the large mixer for an additional 15 min before adding the fish oil, and mixing for a further 15 min. The mixture was extruded (Clextral EV0 A107) at 50 °C, 70 °C, 80 °C, 91 °C and 100 °C, in five consecutive barrels. The pellets obtained were dried O/N at 35 °C in a forced air circulator.

**Table 1:** Dietary formulation of the experimental diets. All the values are in g Kg^−1^

	Acclimatization diet	Diet suppl with 1 mg Se Kg^−1^	Diet suppl with 4 mg Se Kg^−1^
	Control diet		
*Fish meal*	100	100	100
*Wheat meal*	198	199.5	198
*Corn gluten*	190	190	190
*Soya*	160	160	160
*Fish oil*	173	173	173
*Wheat gluten*	174	172	172
Vitamins and minerals*	5	5	5
*Sel-Plex addition (@ ~ 2 mg Se g* ^*−1*^ *)*	-	0.5	2

*Vitamins and minerals composition is provided in Additional file 3: Table S1

Six fish per tank (ie 25 % of total fish used) were weighed at the beginning of the feeding trial, and after two, four, six and eight weeks, and the average body weight was recorded to allow adjustment of the feed quantity given. We have previously shown that at these inclusion levels there is no impact on fish growth (Pacitti et al., 2015). Fish were fed twice daily, an amount equating to 2 % of the average body weight. With fish of this size it is typical to keep the feeding ration constant, as in this study. After ten weeks, ten fish from each tank were injected with PBS and ten with 1.25 mg of poly(I:C) (Sigma-Aldrich). For the poly(I:C) preparation, 100 mg were dissolved in 36 ml of nuclease-free water (Sigma-Aldrich) plus 4 ml of 10X PBS (2.5 mg/ml final poly(I:C) concentration), and 0.5 ml was injected intraperitoneally (i.p.) into each fish. Fish injected with PBS and poly(I:C) were marked differently using a panjet containing 2 % alcian blue (Sigma-Aldrich). Nine fish injected with PBS and nine fish injected with poly(I:C) from each tank were killed 24 h post injection for tissue harvest, a time previously shown by us to be optimal for in vivo gene expression changes following i.p. injection of a PAMP such as poly(I:C) or recombinant cytokines [21–24]. All fish were killed by a schedule 1 method (http://www.nc3rs.org.uk/) in all experiments.

Liver and HK were placed on dry ice and subsequently processed for RNA extraction, and a portion of liver was placed on dry ice and further processed for Se bioaccumulation analysis.

### Se bioavailability from diet and Se bioaccumulation in tissues

For total Se analysis of the diets and trout tissues, 100 mg of the lyophilized pellets/organs (three replicates of each) were weighed in a 50 ml Teflon vessel. 1 ml of nitric acid was added to each vessel containing the samples and allowed to stand overnight before 2 ml hydrogen peroxide was added to the vessel, which was closed to prevent analyte loss. The samples were digested using a CEM mars microwave digester. The samples were brought to 20 ml and analysed using inductively coupled plasma mass spectrometry (ICP-MS). The ICP-MS was operated with a forward power of 1380 W under normal conditions, with nickel sampler and skimmer cones. Carrier gas flow was 1.27 L/min, coolant gas flow 14 L/min and nebuliser gas flow 0.86 L/min. Total Se concentration was determined by monitoring ^77^Se and ^78^Se isotopes by external calibration. Germanium was used as an internal standard. In order to evaluate the accuracy of the method, total selenium content in dogfish muscle (DORM – 2) was measured at the end of each time point analysis and the percentage recovery range determined was between 99.5–102 %.

### RNA isolation

RNA was extracted from 100 mg tissue by homogenization in 1.4 ml TRIzol (Sigma-Aldrich) using a stainless tungsten carbide bead (5 mm, Qiagen) and the TissueLyser II system (Qiagen), at 30 Hz for 3 min. To extract RNA from the homogenate, 300 μl of chloroform was added to the tissue lysates. The aqueous phase containing RNA was transferred to a fresh RNase-free 1.5 ml tube containing an equal amount of chilled isopropanol (Sigma-Aldrich). The RNA pellet was washed in 900 μl 70 % molecular grade ethanol (VWR International), air-dried and dissolved in 20 μl of nuclease-free water.

Total RNA was first quantified by spectrophotometry (ND-1000, NanoDrop) and the integrity of all the samples was determined using RNA Nano Chips and the RNA 6000 Nano Assay Kit (Agilent), with an Agilent 2100 bioanalyzer (Agilent). The RNA was stored at −80 °C until required.

### Quantitative PCR (qPCR)

First strand cDNA was synthesized from 2 μg of total RNA using 1 μl RevertAid™ reverse transcriptase (10,000 U, Fermentas) in the presence of 5 μl 5X Reaction Buffer, 1 μl dNTP (Bioline), made up to a final volume of 25 μl with water and incubated at 42 °C for 2 h.

QPCR was performed with a LightCycler 480 (Roche) to quantify the expression of selected selenoprotein transcripts and a set of common reference genes using the primers given in Table 2. The primers employed for qPCR were designed with at least one primer across a predicted intron and pre-tested to ensure that each primer pair could not amplify genomic DNA using the qPCR protocols. The qPCRs were performed in duplicate for each sample, along with a 10-fold serial dilution of references consisting of an equimolar mix of purified PCR products of each gene amplified from cDNA. The transcript level was calculated using the quantitative fit points method in the integrated LightCycler 480 software. Thus, the relative expression level of the candidate genes in different tissues was expressed as arbitrary units, which were calculated from the serial dilution of references run in the same plate and then normalised against the expression level of the house-keeping genes. The target gene expression was normalised against the geometric mean of the expression of the house-keeping genes elongation factor 1α (*ef1a*), DNA directed RNA polymerase II subunit I (*drpII*) and hypoxanthine phosphoribosyltransferase 1 (*hprt1*).

**Table 2:** Primers used for *q*PCR

Gene name	Primer name	Primer sequence (5’ → 3’)	Product size	Acc Number
*Elongation factor-1α*	EF1α-F	CAAGGATATCCGTCGTCGTGGCA	327	AF498320
	EF1α-R	ACAGCGAAACGACCAAGAG		
*DNA directed RNA*	DRPII-F	TCACCCATGAAGTTGATGAGCTGA	176	BT073753
*polymerase III subunit I*	DRPII-R	CCGTGCAGACATAGTACAGCCTCA		
*Hypoxanthine*	HPRT1-F	GCCTCAAGAGCTACTGCAATG	256	ACH70616
*Phosphoribosyltransferase 1*	HPRT1-F	GTCTGGAACCTCAAATCCTATG		
*Thioredoxin reductase 3a*	TrxR3a-F	AGTCAACCCCAAGAACGGTAAGG	297	HF969246
	TrxR3a-R	CAGAAGAGACTGTGGTACACCTCCAA		
*Thioredoxin reductase 3b*	TrxR3b-F	CAAAGTCAACCCCAAGAATGGTAAGA	300	HF969247
	TrxR3b-R	CAGAAGAGACTGTGGTACACCTCCAG		
*Selenoprotein Pa*	SelPa-F	GCTTGGTGCAGGCATCCTTATTG	276	HF969249
	SelPa-R	CATATCTCCCTGCCCTACTCCATCC		
*Selenoprotein Pb*	SelPb-F	GACGACTTCCTGGTATATGACAGATGTG	275	HF969250
	SelPb-F1	GATACCGTCAGCAACCCAGTTCC		
*Interferon 1a*	IFN-1aF	CTGTTTGATGGGAATATGAAATCTGC	193	AJ580911
	IFN-1aR	CCTGTGCACTGTAGTTCATTTTTCTCAG		
*Interferon 1b*	IFN-1bF	GATGGGAATAGGAATAGGAATAGGAAGTC	200	AJ582754
	IFN-1bR	GCCTCTGCACTGTAGTTCATTTTTCTC		
*Interferon γ1*	IFN-γ1F	CAAACTGAAAGTCCACTATAAGATCTCCA	210	AJ616215
	IFN-γ1R	TCCTGAATTTTCCCCTTGACATATTT		
*Interferon γ2*	IFN-γ2F	CAAACTGAAAGTCCACTATAAGATCTCCA	188	FM864345
	IFN-γ2R	GGTCCAGCCTCTCCCTCAC		
*Interferon γ induced protein*	CXCL11-F	CATCAGCTTCCTGGCCTGTC	187	AJ417078
	CXCL11-R	CCGTTCTTCAGAGTGACAATGATCTC		
*Viperin*	VIG-F	AGAACTCAACCCTGTACGCTGGA	227	AF076620
	VIG-R	GGCAATCCAGGAAACGCATATATTC		
*Laboratory of genetics*	LGP2-F	CAGGGACTTCCGAATGAAGATCAC	230	FN396358
*and physiology 2*	LGP2-R	CGCCGGTCTTATAGTACCTCTCAAAGTC		
*Melanoma differentiation-*	MDA5-F	CCTTTTCACGCTCTTTAAAGATAGCAAAG	229	FN396357
*associated gene 5*	MDA5-R	GCAAGCTTTTACTCCGACCTCCTC		
*Toll-like receptor 3*	TLR3-F	GAACGTTCTGATCAACCGTACGCT	297	AY883999
	TLR3-R	TGGGCCATGAACTGTCGACA		
*Toll-like receptor 9*	TLR9-F	GGCTGCTGGATGAAAAGGTGGA	212	EU627195
	TLR9-R	CTCGTTGACGTTGCTGTCGTAGGA		
*MX protein 2*	MX2-F	CCTTCTGAAAACAGCAAAGACTAAGA	184	OM47945
	MX2-R	AACTAACTCTCCCTCCTCCAACTC		
*Serum amyloid A*	SSA-F	GGTGAAGCTGCTCAAGGTGCTAAAG	162	AM422447
	SSA-R	GCCATTACTGATGACTGTTGCTGC		
*Suppressor of cytokine*	SOCS3-F	CACAGAGAAACCGTTAAAAGGACTATCC	228	AM748723
*signalling 3*	SOCS3-R	AAGGGGCTGCTGCTCATGAC		
*Cathelicidin 1*	CATH1-F	ACCAGCTCCAAGTCAAGACTTTGAA	275	AY594646
	CATH1-R	TGTCCGAATCTTCTG CTGCAA		
*Hepcidin*	HAMP-F	GCTGTTCCTTTCTCCGAGGTGC	165	CA369786
	HAMP-R	GTGACAGCAGTTGCAGCACCA		
*Insulin-like growth factor*	IGFBP-1b1F	ATCCCAGACCCCTCCACTCC	258	JX565545
*binding protein*	IGFBP-1b1R	GCTGAGAGCTGGTTATCTTGTCC		

### Microarray

The transcriptomic response of both HK and liver to poly(I:C) stimulation after Sel-Plex supplementation was measured. A preliminary qPCR screening was carried out to determine which of the two groups fed supra-nutritional levels of Sel-Plex showed a more significant response to the treatment. The expression of selected selenoprotein transcripts and mediators of the antiviral response were measured, and based on these results one of the two experimental diet groups was selected to compare against the control.

The experimental design (Additional file 1: Figure S1) was a common reference design for which the reference sample was an equal mixture of all the experimental samples and antisense RNA (aRNA) from the experimental groups was hybridized against this common control aRNA sample. The experimental samples were a pool of equal amounts of RNA extracted from three different fish fed a control diet or a diet enriched with 4 mg Se Kg^−1^, and injected with PBS or poly(I:C) (a total of four experimental groups), with each fish from one of the three tanks assigned per diet. The RNA was pooled prior to the aRNA amplification. The aRNA was amplified using a MessageAmp™ aRNA Amplification Kit (Ambion). Briefly, 2 μg of total RNA was reverse transcribed and the cDNA was used as a template for *in vitro* transcription in the presence of amilo allyl modified dUTP, which allowed the generation of amplified aRNA. For labelling, 3 μg aRNA was denaturated at 70 °C for 2 min in a volume of 10 μg to which 3 μl of 0.5 M NaHCO_3_ (pH 9.0) and a 2 μl aliquot of Cy dye (Dye Cy3™ and Cy5™ mono-reactive dye pack, Amersham PA23001-PA25001) was added. Incorporation of dye was performed for 1 h at 25 °C in the dark, and afterwards excess label was removed using a DyeExTM 2.0 spin kit (QIAGEN). The quantity of each labelled dye was checked using spectrophotometry (ND-1000, NanoDrop), and the labelled aRNA was either hybridized immediately or stored at −80 °C.

The microarray common control was created by mixing equal amounts of aRNA from every sample included in the experiment. The common control was labelled with Cy5, whereas the samples were labelled with Cy3 (described below). The four experimental groups were distributed on the four arrays of an Agilent 4x44K slide, with different replicates across slides. A total of five slides were used for each organ (HK and liver). However one slide for the HK was lost due to a scanner fault, whereas one array from one slide for liver was lost due to a dye signal issue.

Prior to hybridization, 825 ng of Cy3 labelled aRNA from each sample and 825 ng of Cy5 labelled aRNA from the common control were mixed in a 1.5 ml tube. The labelled template was fragmentated in the presence of 11 μl of 10X Blocking Agent (Agilent) and 2.2 μl of 25X Fragmentation buffer (Agilent), brought to a final volume of 20 μl with nuclease-free H_2_O and incubated in the dark at 60 °C for 30 min. Subsequently the solution was cooled on ice for 1 min and then 57 μl of 2X GEx Hybridization buffer (Agilent) was added to the mix to stop the fragmentation reaction. Immediately afterwards 103 μl of each hybridization solution was dispensed onto the Agilent 4x44K gasket slides. Next the rainbow trout “*Trout 2013*” oligo array was placed onto the gasket slide and the “sandwich” placed in the hybridization chamber. The hybridizations were performed in a Microarray Hybridization Oven (Agilent) overnight (18 h) at 65 °C. For washing, the slides were rinsed using two different wash solutions: Gene Expression wash buffer 1 (Agilent) and Gene Expression wash buffer 2 (Agilent). Prior to the washing 0.005 % Triton X-102 (Agilent) was added to the Gene Expression wash buffers to reduce the possibility of array wash artefacts. The slides were then scanned on an Axon 4200A scanner (Axon Instruments) at a resolution of 5 μm and the images saved as *.TIF files.

Images were extracted and initial analysis was performed by Feature extraction v9.5.3 (Agilent), performing background correction of feature intensities (within the software). A Lowess normalisation of background corrected data was next conducted and all intensity values <1.0 were set to 1.0.

The trout array used in this study (“*Trout 2013*”) was updated with all trout cDNA sequences published within the NCBI gene database and all the trout selenoprotein sequences cloned in our previous work [25, 26] were included. Probes matching sequences for selenoproteins or molecules involved in selenoprotein synthesis (*e.g.* DIOs, Fep15, SelJ, SelK, SelL, SelP, SelR, SelS, SPS2 and EFsec) retrieved *in silico* were added to the array in duplicate. All the non-annotated sequences and all the ones having an *E* > 0.001 when using blast with the tBLASTx function at NCBI gene bank were excluded. Furthermore, all the duplicate probes matching to the same target were eliminated with a similar approach. A unique probe per target was chosen based on the *E* value given after tBLASTx within NCBI gene bank; probes with the same identity for a unique target were further selected based on their identity with the corresponding human orthologue after BLASTx within the Ensemble human genome database http://www.ensembl.org/index.html), and an official genome symbol was assigned using the HUGO Nomenclature Committee (HGNC, http://www.genenames.org/).

### Statistical analysis

Data from ICP-MS and *q*PCR are presented as means + S.E.M., plotted using GraphPad Prism software (V5), and analyzed using the SPSS package 21.0 (SPSS Inc. Chicago). Significant differences were indicated at *p* < 0.05 using one way-analysis of variance (ANOVA) and Tukey's post hoc test, or two-way analysis of variance (ANOVA).

Statistical analysis of the arrays was performed using the Genespring GX analysis platform (version 9.5; Agilent Technologies). Quality control of the data was performed within Genespring and included removal of saturated probe features, non-uniform features, population outliers and those features showing intensities not significantly different from background in the Cy3 or Cy5 channels.

After these relatively stringent procedures, a number between 25,000 and 30,000 of the original 45,220 array features were maintained for subsequent analyses. The Genespring statistical tool was used to analyse the differences amongst the treatment groups included in the microarray experiment. Benjamini and Hochberg False Discovery was the correction applied to the data, with significant differential expression established by either one-way ANOVA (*p <* 0.05) followed by Tukey's post hoc test or two-way ANOVA (*p <* 0.05), depending on the kind of comparison being made. Further filtering on fold change was conducted and only transcripts showing ≥2 fold change in expression were considered further. The experimental hybridisations are at the European Bioinformatics Institute, archived under accession number E-MTAB-2982.

### Gene ontology (GO) analysis

For enrichment analysis of biological process ontology (gene ontology, GO), the up- and down-regulated transcripts were analysed as two dependent clusters within ClueGO, a Cytoscape plug-in that visualizes the non-redundant biological processes in a functionally grouped network [27]. The HGNC symbols corresponding to the up- and down- regulated trout transcripts from each comparison list of interest were inputted into ClueGO and a minimum of three genes was used as cut-off to find the GO term. The two-sided hypergeometric method was used for statistical testing and the *p* values were corrected with the Bonferoni step-down method. Only terms with a *p <* 0.05 are shown. A Kappa score equal to four was used as the cut-off for GO term grouping for the network analysis.

## Results

### Se bioaccumulation and preliminary PCR screening

**Fig. 1:**
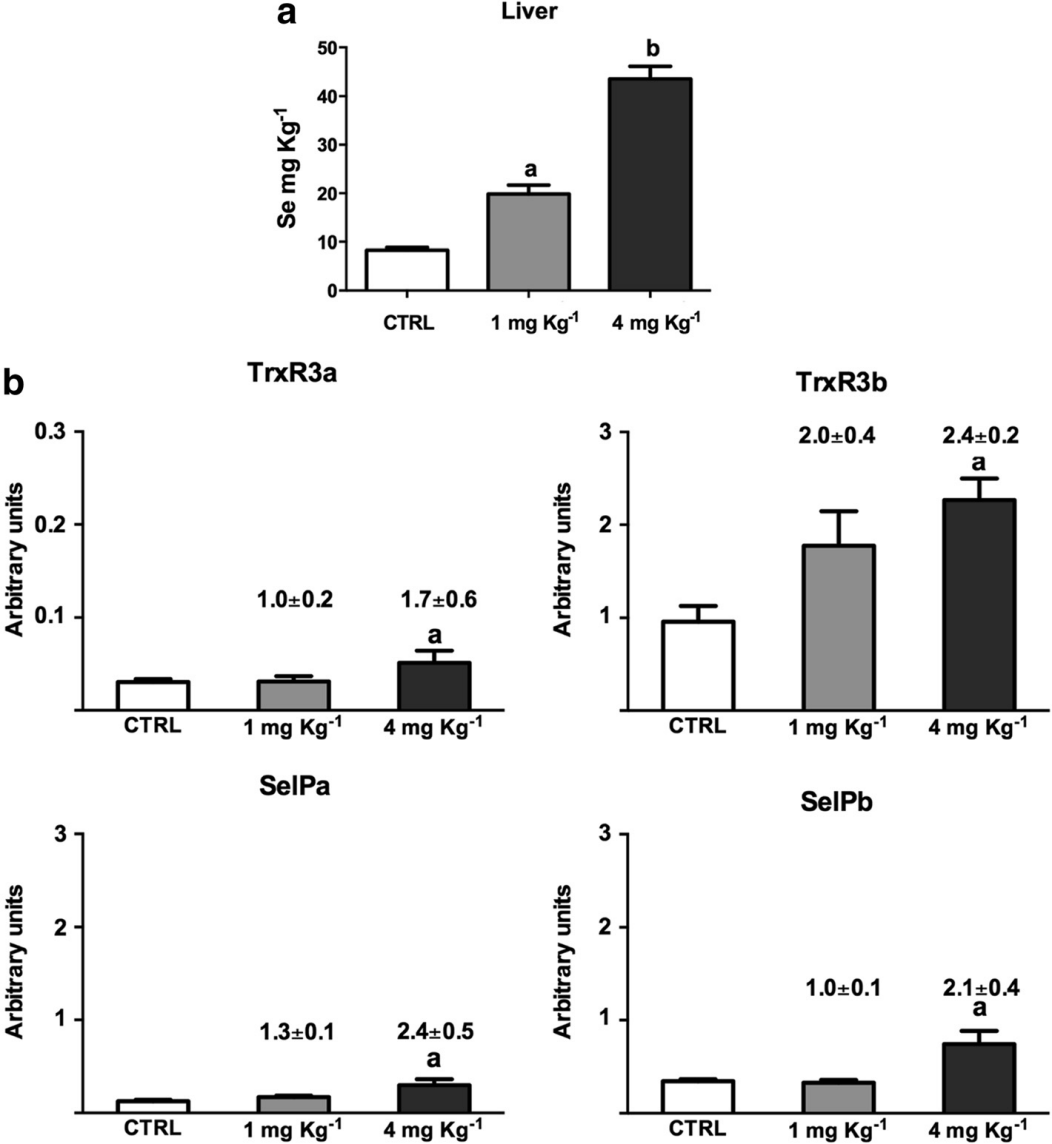
Se concentration in liver (**a**), and transcriptional modulation of selected selenoprotein genes in liver (**b**). Total Se concentration was determined in liver tissue using ICP-MS in reaction cell mode. The results represent the mean + SEM of 12 fish from three different tanks for each diet group. The expression of gene transcripts was quantified by *q*PCR and normalized against the geometric mean of three housekeeping genes (*ef1α*, *drpII*, *hprt1*), and then used for statistical analysis. The transcript expression is reported as arbitrary units and a fold change, calculated as the average expression level of fish fed the control diet divided by that of fish fed the experimental diets, is reported above the bars. The results represent the mean + SEM from 18 fish from three replicate tanks for each experimental group. The letters above the columns indicate statistically significant results versus the controls, as assessed by one-way ANOVA (*p* < 0.05), with different letters indicating significant differences between the treatments

Se concentration in the experimental diets and its bioaccumulation in liver tissue were determined using inductively coupled plasma mass spectrometry (ICP- MS) (Fig. 1a). The Se concentration in the control diet was equal to 1.10 ± 0.05 mg Kg^−1^, likely due to the animal source components of the diet (i.e. fish meal and fish oil). Thus the expected Se concentration in the supplemented diets was 2.1 and 5.1 mg Kg^−1^ and when the Se in these two diets was measured the Se content was found to be 2.61 ± 0.09 and 6.35 ± 0.16 mg Kg^−1^ respectively. In the liver of fish fed the control diet, 8.3 ± 0.5 mg Kg^−1^ of Se was bioaccumulated, whereas 19.9 ± 1.8 and 43.5 ± 2.6 were detected in the groups fed the 1 and 4 mg Se Kg^−1^ enriched diets respectively. From the Se results, it is possible to assert that Se can be efficiently and proportionally assimilated in the diets. No impact on fish health was noted, and no deaths were recorded during the feeding trial. There were no significant differences in average body weight between the three groups at the end of the experiment.

Next, we measured in liver the transcript expression of *trxr3* and *selp* (selenoprotein P) isoforms (Fig. 1b), as indicators for Se bioassimilation in trout [20]. All the selected transcripts showed a significant induction in the group fed 4 mg Se Kg^−1^.

**Fig. 2:**
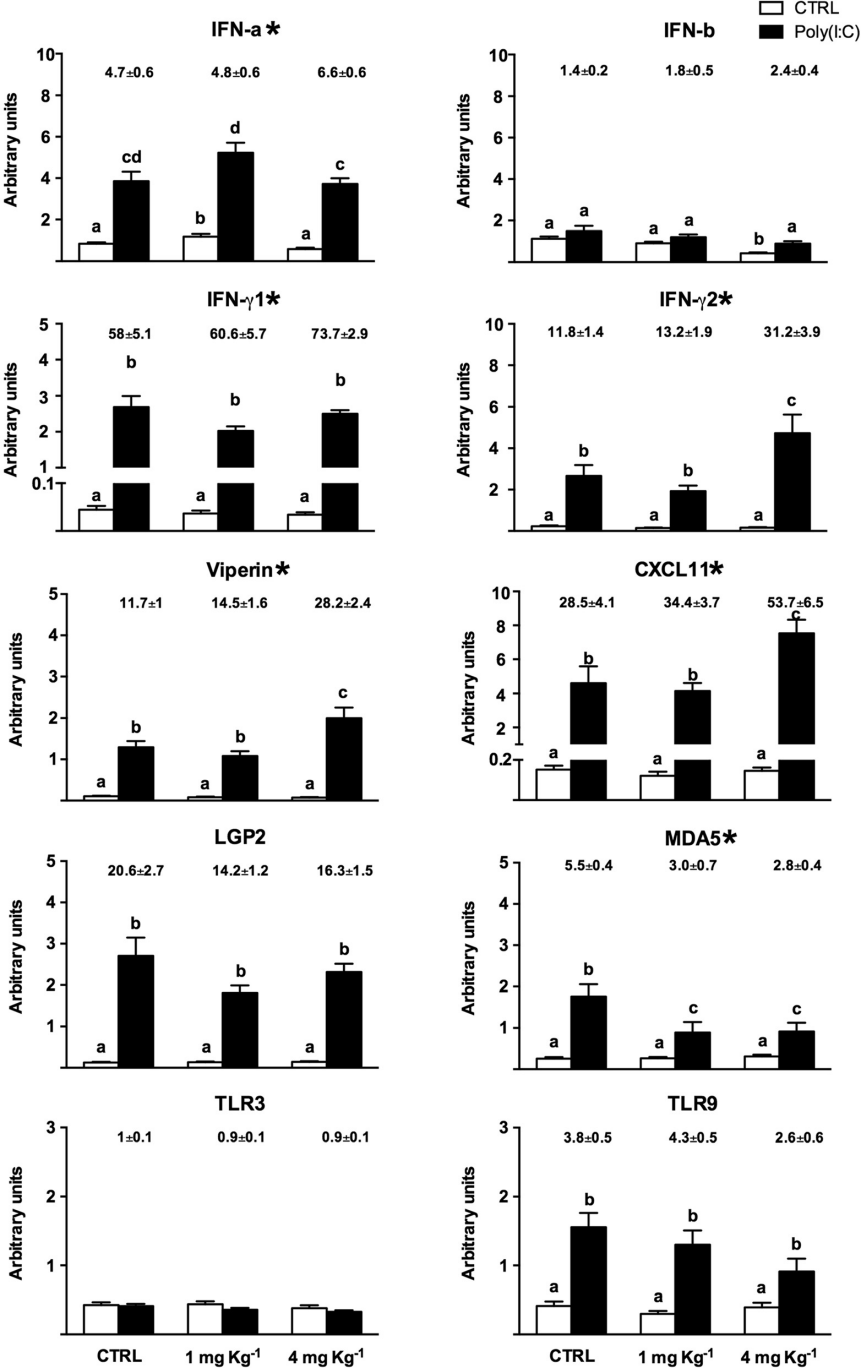
Transcriptional modulation of selected antiviral mediators in HK. After 10 weeks of feeding, the fish were injected with vehicle (PBS) or poly(I:C) (2.5 mg/ml) and 24 h later tissues were harvested. The expression of gene transcripts was quantified by *q*PCR and normalized against the geometric mean of three housekeeping genes (*ef1α*, *drpII*, *hprt1*), and then used for statistical analysis. The transcript expression is reported as arbitrary units and a fold change, calculated as the average expression level of fish injected with poly(I:C) divided by that of fish injected with PBS, is given above the bars. The results represent the mean + SEM from 27 fish from three replicate tanks for each experimental group. The letters above the columns indicate values that are statistically significant versus the controls assessed by one-way ANOVA (*p* < 0.05), with different letters indicating significant differences between the treatments. The asterisks indicate the transcripts that show a significant interaction between poly(I:C) stimulation and Se supplementation, as assessed by two-way ANOVA

Furthermore, we analysed the differential response of selected antiviral response mediators in the HK of fish fed the experimental diets and subsequently stimulated with poly(I:C) (Fig. 2). Upon inspection of the interferon (IFN) gene response, a differential trend was observed for type I IFN (predominantly *ifn-a* and *ifn-b*) and type II IFN (*ifn-γ*) in the two groups fed diets enriched with 1 and 4 mg Se Kg^−1^. In the PBS injected individuals, the *ifn-α* transcript was up-regulated after feeding the diet with the lower concentration of Sel-Plex but was unchanged with the other experimental diet. A similar trend was seen after poly(I:C) stimulation, with the group fed the diet enriched with 1 mg Se Kg^−1^ showing a higher induction of *ifn-α*. The interaction of the lower Sel-Plex augmentation and the *ifn-a* response to poly(I:C) stimulation was further confirmed by two-way ANOVA analysis as shown in Fig. 2.

*Ifn-b* transcript expression was instead inhibited by increasing concentration of Sel-Plex, with a significant down-regulation detected in the group supplemented with 4 mg Se Kg^−1^ and injected with PBS. In contrast, *ifn-γ* isoforms both showed a significant interaction between Se supplementation and response to poly(I:C), primarly in the group fed the diet enriched with 4 mg Se Kg^−1^. The higher fold change for *ifn-γ* isoform 1 expression was likely due to a slight but not significant inhibition of the same transcript due to the experimental diet, whereas with isoform 2 a strong induction in response to the poly(I:C) was seen regardless of the response in the vehicle group. For both isoforms of *ifn-*γ, a strong interaction between the dietary treatment and immunological response was further confirmed statistically. Viperin and CXCL11 transcripts, two important mediators of the IFN response, showed a similar pattern of expression to that of *ifn-γ* isoform 2, with a significantly higher up-regulation in the group fed the diet enriched with 4 mg Se Kg^−1^.

Finally the expression of receptors involved in antiviral sensing were examined, including relevant toll-like receptors (TLR) and members of the retinoic acid-inducible gene I (RIG-I)-like helicase (RLH) family. The transcript expression of melanoma differentiation-associated gene 5 (MDA5) and laboratory of genetics and physiology 2 (LGP2) genes was not affected by Sel-Plex supplementation, and only the *mda5* transcript showed a significant interaction between poly(I:C) stimulation and Se supplementation, as assessed by two-way ANOVA. *Tlr3* transcript expression was also not modulated by either treatment, whereas *tlr9* mRNA was induced by poly(I:C) in all these groups.

### Overview of transcriptomic response analysis

From the preliminary *q*PCR screening, modulation of the transcript expression for selected selenoproteins and antiviral mediators was mostly seen in the group fed a diet enriched with 4 mg Kg^−1^ Sel-Plex. Therefore this diet group was selected to be compared to the control group within the microarray experiment.

Several transcripts in the HK showed a differential response to poly(I:C) between the control diet group and the fish fed a diet enriched with 4 mg Se Kg^−1^, whereas transcripts within the liver were altered by the diet.

To examine the overall effects of poly(I:C) and Sel-Plex supplementation on gene transcription, signal intensity values of all probes with significant expression were subjected to principle component analysis (PCA) (Additional file 2: Figure S2). The PCA results indicated that within the HK there is an interaction between the immune challenge and the diet, likely due to a large effect of the experimental diet on transcript expression within this tissue. However, in the liver there was an effect of both the poly(I:C) injection and Sel-Plex supplementation but no interaction was found.

**Fig. 3:**
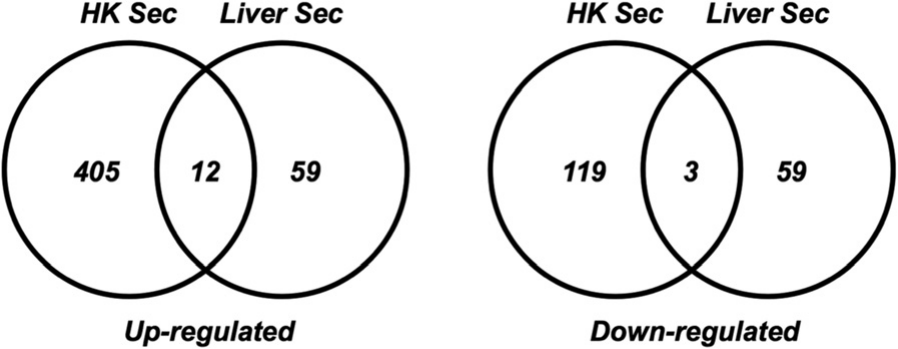
Genes expressed at different levels in HK and liver upon dietary supplementation with Sel-Plex. The Venn diagram shows the genes identified by microarray analysis, differentially expressed in HK and liver from trout fed a diet supplemented with 4 mg Se Kg^−1^ relative to the group fed a control diet. All the genes presented as up-regulated and down-regulated are significantly altered in expression as tested by one-way ANOVA and Tukey’s HSD multiple comparisons test (*p* < 0.05 and Benjamini-Hochberg correction) with ≥2 fold change in expression

**Fig. 4:**
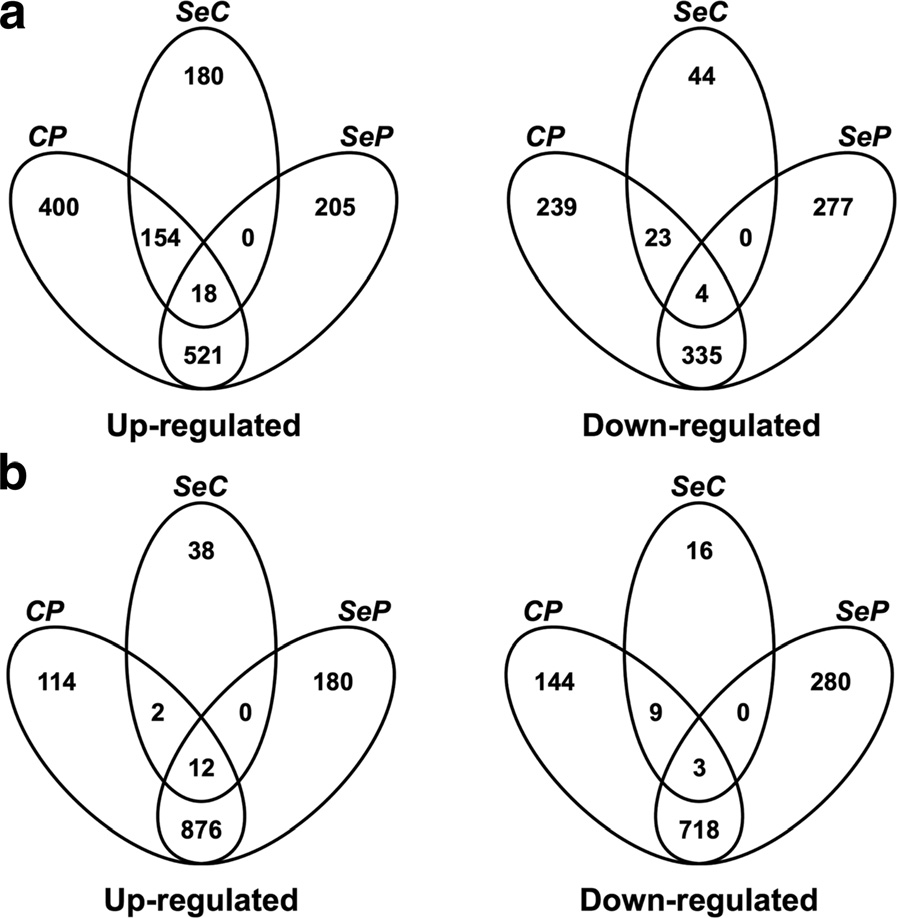
Genes expressed at different levels in HK (**a**) and liver (**b**) upon dietary supplementation with Sel-Plex and immune challenges. The Venn diagram shows the genes identified by microarray analysis significantly altered in expression as tested by one-way ANOVA and Tukey’s HSD multiple comparisons test (*p* < 0.05 and Benjamini-Hochberg correction), with ≥2 fold change in expression. The genes up-regulated and down-regulated in the group fed the 4 mg Kg^−1^ Sel-Plex enriched diet relative to the group fed a control diet are named SeC. The genes up-regulated and down-regulated in the group fed a control diet or a 4 mg Se Kg^−1^ enriched diet and injected with poly(I:C), relative to the same diet groups injected with PBS, are indicated as CP and SeP respectively

Overall, the challenges resulted in large global alterations in transcriptional activity; 5063 probes were detected as being significantly modulated in the HK and 5596 in the liver, by the two treatments. Looking specifically at the diet effects, it is evident that Sel-Plex had a larger effect on the HK than on the liver (Fig. 3), further confirming a strong effect of supplementation on this tissue. A list of 2409 and 2385 transcripts that were significantly modulated with a fold change ≥ 2 in at least one of the contrasts of interest in the HK and liver, respectively, was obtained (Fig. 4).

Due to the large transcriptomic response encountered in both tissues, we first determined the main biological pathways involved in this response before examining the modulated transcripts, looking into the most significant processes highlighted by the gene ontology (GO) analysis. To this end, the up- and down-regulated transcripts were analysed as two dependent clusters within ClueGO, a Cytoscape plug-in that visualizes the non-redundant biological processes in a functionally grouped network [27].

The microarray output was confirmed by real time *q*PCR analysis, measuring the transcript expression of genes encoding for components of the immune response and selenoproteins in the three comparisons of interest that were selected (Additional file 4: Table S2). For all genes the expression pattern showed the same direction of response between microarray and *q*PCR analysis albeit the magnitude of the expression differences varied between the two platforms.

### Liver transcriptomic response to Sel-Plex supplementation

**Fig. 5:**
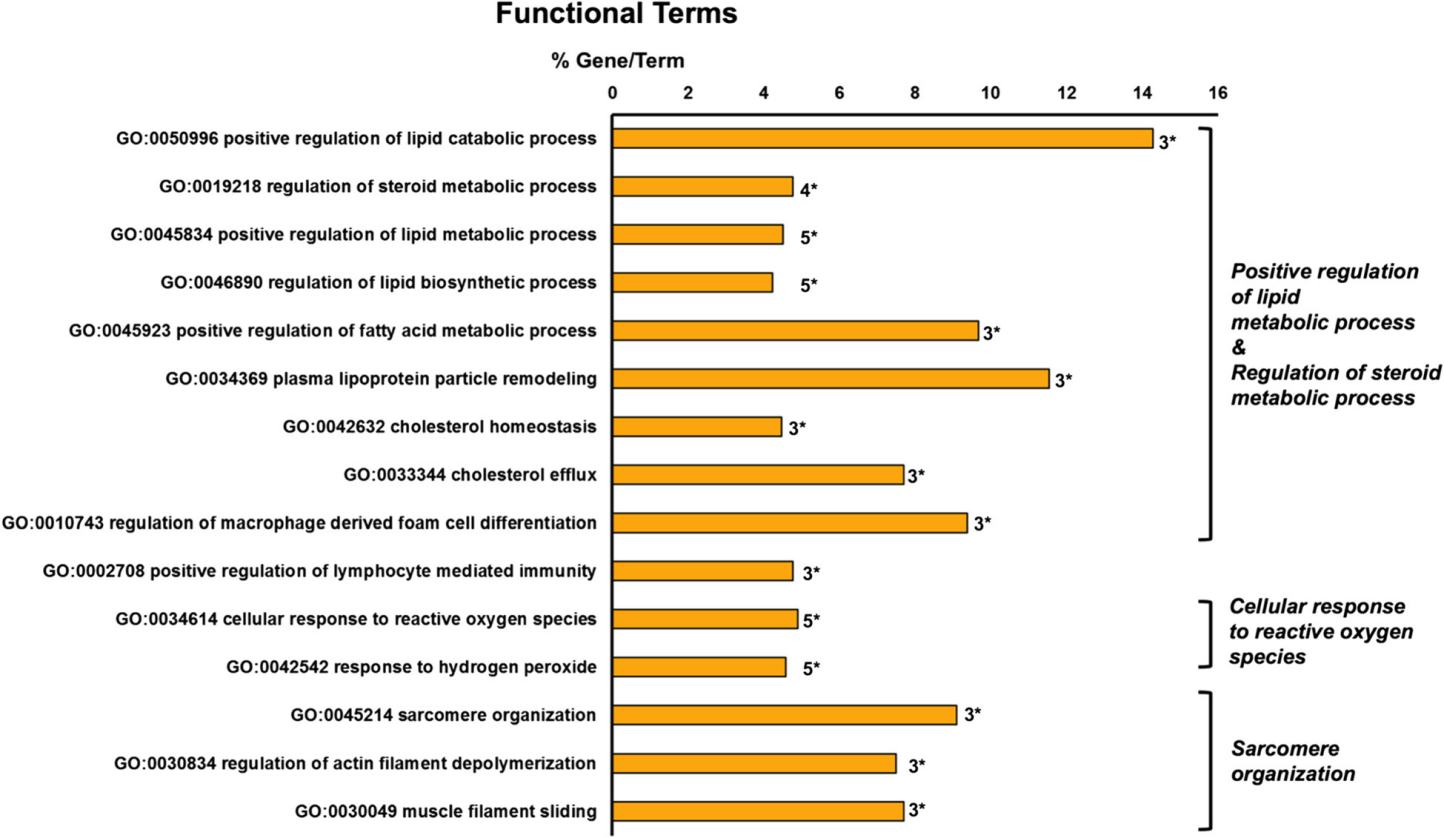
Functional terms enrichment of the identified transcripts in the liver of fish fed a diet enriched with 4 mg Se Kg^−1^. The bars represent the percentage of genes found compared to all the genes associated with the term, and the number of genes is displayed. The minimum number of genes assigned to each term was three. A two-sided hypergeometric method was applied as the statistical test and the *p* values were corrected with the Bonferoni step-down method. Only terms with a *p* < 0.05 are shown. A Kappa score equal to four was used as cut-off for GO term grouping. The level of significance for terms and groups is indicated with an asterisk: “*”*p* < 0.05

In the liver, 133 transcripts were significantly altered when comparing the response to the experimental diet of this tissue and HK (Fig. 3). Among these, only 20 transcripts were found to be involved in significant GO terms (Table 3), and due to the size of the gene cluster it was not possible to determine clearly between the up- and down-regulated transcript clusters as to which had the major contribution to the biological processes found to be modulated. The main biological pathways highlighted by the GO analysis were processes involved in the regulation of lipid metabolism, more specifically steroids, lymphocyte mediated immunity, response to ROS and sarcomere organization (Fig. 5).

**Table 3:** List of selected transcripts significantly modulated in the liver of fish fed the Sel-Plex enriched diet^2^

Trait	SeC	SeC	SeP	CP	Acc.	Identity7	HGNC	Gene Function^9^
Identifier^1^	Liv^2^	HK^3^	Liv^4^	Liv^5^	Number^6^		symbol^8^	
TC147106	4.1				BT048822	Apolipoprotein A-IV precursor	APOA4	positive regulation of lipid catabolic process
								plasma lipoprotein particle remodeling
TC143041	2.4	2.4	−2.2	−1.3	EF062859	cAMP responsive element-binding protein	CREB1	positive regulation of lipid metabolic and biosynthetic process
TC162807	−3.2				AF281344	Fatty acid binding protein	FABP1	positive regulation of lipid catabolic process and fatty acid metabolic process
TC132505	−2.9		−1.7	−1.4	NM_001124594	Growth hormone secretagogue receptor 1a	GHSR	positive regulation of fatty acid lipid metabolic process
TC133989	−2.0				AJ223954	Interleukin-1-beta	IL1B	positive regulation of lipid catabolic process and lymphocyte mediated immunity
TC161770	2.3				DQ789367	nonclassical MHC class I antigen	MR1	positive regulation of lymphocyte mediated immunity
TC167571	2.1				NM_001165374	Lysosomal-associated membrane protein 1	LAMP1	positive regulation of lymphocyte mediated immunity
TC134954	2.0				not-annotated	not-annotated	CRP	regulation of macrophage derived foam cell differentiation
TC154249	−2.1				DQ156149	Salmo salar clone BAC S0085O16	CETP	plasma lipoprotein particle remodeling
								regulation of macrophage derived foam cell differentiation
TC132491	−2.6				AY600084	Vitellogenin 2	APOB	plasma lipoprotein particle remodeling
								regulation of macrophage derived foam cell differentiation
TC172388	2.6		−2.3	1.3	NM_001124329	Superoxide dismutase 1 soluble	SOD1	regulation of steroid metabolic process
								cellular response to reactive oxygen species
TC169043	2.3				NM_001160555	Hemoglobin subunit beta-1	HBB	cellular response to reactive oxygen species
TC133128	−2.8				NM_001124660	Cyclin-dependent kinasekinase	CDK1	cellular response to reactive oxygen species
TC149187	−2.3				EU221177	Salmo salar clone 242 N16	LRRC16A	regulation of actin filament depolymerization
TC136960	−2.4				NM_001140990	Vacuolar protein sorting 72 homolog	VPS72	regulation of actin filament depolymerization
TC168877	−2.1				NM_001165377	Beta-adducin	ADD2	regulation of actin filament depolymerization
TC156862	−2.4				NM_001124352	Type II keratin E2	KRT8	sarcomere organization
TC137650	−3.1	−4.2	1.3	−1.1	BT125454	Telethonin	TCAP	sarcomere organization
								muscle filament sliding
TC163493	2.2		−1.2	1.6	NM_001139606	Slow troponin T family-like	TNNT2	sarcomere organization
								muscle filament sliding
TC134405	−2.2		−1.7	−3.0	BT049717	Myosin light polypeptide 3	MYL4	muscle filament sliding

The selection was based on the results of the GO analysis (Fig. 5). Genes with corresponding microarray feature code^1^ involved in a biological process that were significantly altered by the experimental diet in the liver were selected. If the transcripts were also significantly modulated in the HK of fish fed the experimental diet and injected with PBS^3^, in the liver of fish fed a normal diet and injected with poly(I:C)^4^, or in the same tissue of fish fed the experimental diet and injected with poly(I:C)^5^, these values are given. All the transcripts shown were significantly modulated at *p* < 0.05 following a Benjamini–Hochberg correction and had a fold change ≥2. Accession numbers of the cDNA sequences^6^, their identity^7^ and the corresponding human orthologue^8^ determined by BLASTx within the Ensemble database are reported. For each gene the function assigned by ClueGO software is also indicated. SeC represents the groups comparison addressed to analyse the effects of the Sel-Plex enriched diet. CP and SeP instead represent the comparisons addressed to analyse the effect of poly(I:C) stimulation on fish fed either a control diet or the experimental diet respectively

The transcripts for Apolipoprotein A-IV (APOA4), cAMP responsive element-binding protein (CREB1) and superoxide dismutase 1 (SOD1) were the up-regulated targets involved in lipid/steroid metabolism; whereas fatty acid binding protein (FABP1), growth hormone secretagogue receptor (GHSR), interleukin 1-*β* (IL-1*β*) and vitellogenin 2 (associated to human apolipoprotein B, APOB) were down-regulated. Vitellogenin in fish is mainly involved in reproduction, and its production might be affected by lipid intake and metabolism [28]. C-reactive protein (CRP) and cholesteryl ester transfer protein (CETP) mRNA were respectively up- and down-regulated, and both are involved in macrophage differentiation to foam cells. SOD1, together with haemoglobin *β* (HBB) were found to positively contribute to the response to ROS, whereas trout cyclin-dependent kinase 2 (corresponding to human cyclin-dependent kinase 1) was inhibited. A trout sequence corresponding to the gene encoding for major histocompatibility complex class I-related (MR1) in humans and lysosomal-associated membrane protein 1 (LAMP1) were also induced and are primarily involved in lymphocyte mediated immune processes together with IL-1*β*. The transcripts for a few genes involved in sarcomere organization were significantly down-regulated: namely *β*-adducin (corresponding to human adducin 2 (beta), ADD2), myosin alkali light chain (specifically myosin, light polypeptide 4, alkali; atrial, embryonic1, MYL4), vacuolar protein sorting 72 homolog (VPS72), type II keratin E2 (similar to human keratin 8, KRT8) and two sequences matching to the human leucine rich repeat containing 16A (LRRC16A) and Telethonin (TCAP) respectively, the latter transcript was also down-regulated in the HK by the diet. Within the same GO term, only the mRNA level of troponin T type 2 (TNNT2) was found to be positively correlated.

The transcript for *Apoa4* was the most up-regulated by Sel-Plex supplementation. In mice, it is known to enhance triglyceride secretion from the liver, preventing over-accumulation of lipids, which can cause toxic effects in this organ [29]. *Apoa4* expression might be correlated with the induction of *creb1*, a gene involved in adipocyte differentiation [30], and together they promote a positive regulation of lipid metabolism.

The induction of *sod1* and *hbb* transcription, both involved in the cellular response to ROS, may be one potential side effect due to Se over-accumulation.

Amongst the biological pathways listed above “lipid/steroid metabolism” was the most predominant process altered by the diet in liver. However, due to the relatively small number of genes found modulated in the liver, it was not possible to perform a deeper functional analysis on the interactions of these biological processes.

### HK transcriptomic response to Se supplementation

**Fig. 6:**
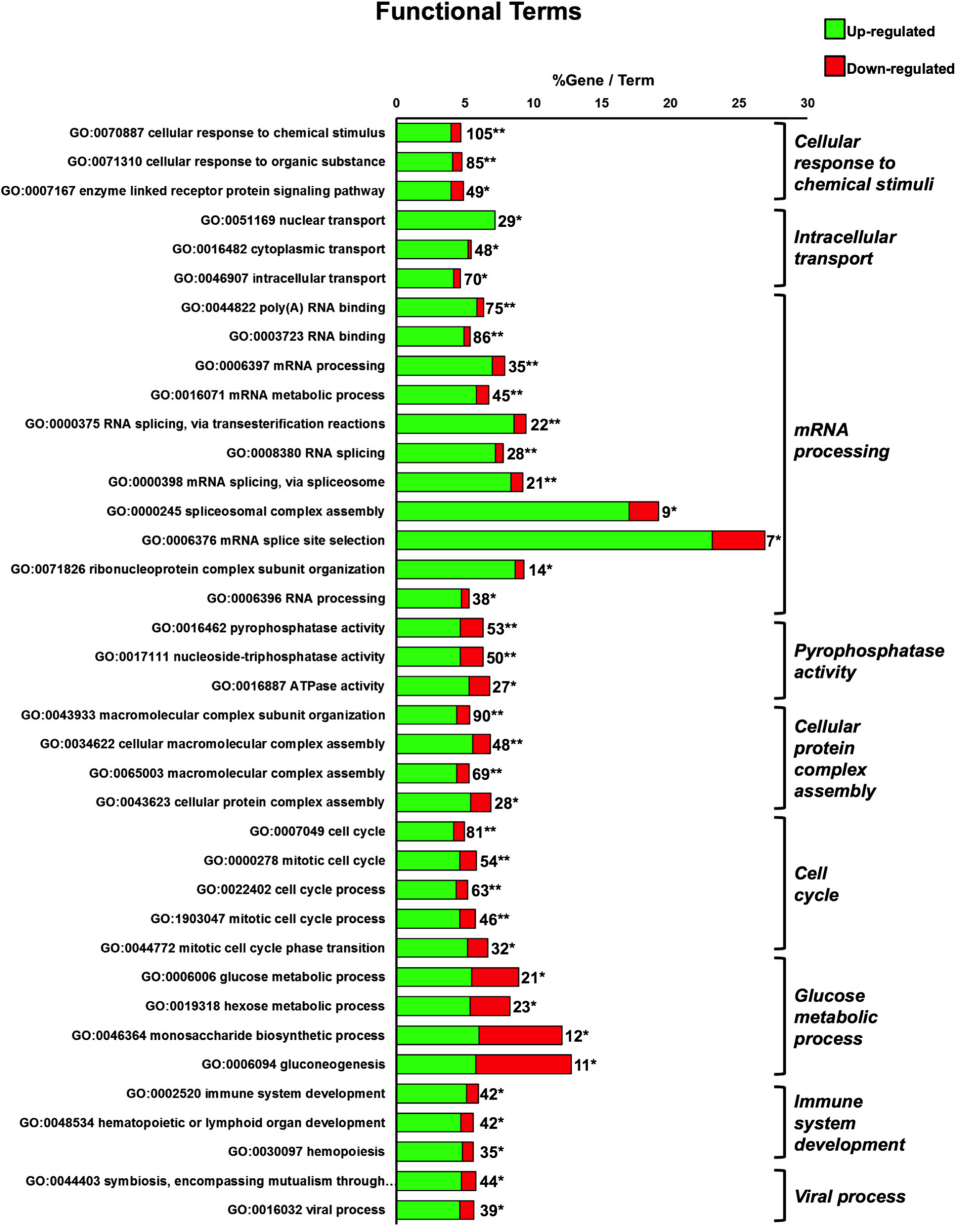
Functional terms enrichment of the identified transcripts in the HK from fish fed the diet enriched with 4 mg Se Kg^−1^. The bars represent the percentage of genes found compared to all the genes associated with the term, and the number of genes is displayed. The minimum number of genes assigned for each term was three. A two sided hypergeometric method was applied as the statistical test and the *p* values were corrected with the Bonferoni step-down method. Only terms with a *p* < 0.05 are shown. A Kappa score equal to four was used as cut-off for GO term grouping. The level of significance for terms and groups is indicated with an asterisk(s): “*”*p* < 0.05 and “**”*p* < 0.001

In the HK, 539 transcripts were significantly altered when comparing the response of this tissue to the experimental diet (Fig. 3). Among these, 407 transcripts were associated with significantly enriched GO terms, of which 322 were up-regulated and 85 were down-regulated. Analysing the two clusters of up/down regulated genes as two distinct but interacting groups, several biological pathways and molecular functions were modulated in the HK upon Sel-Plex supplementation, and all of them were mostly due to the over-expression of several transcripts. All the significant biological processes and molecular functions are listed in Fig. 6, and subdivided under the term considered most appropriate and descriptive for the entire group. Due to the dimension of the set of transcripts found modulated within this contrast, the gene clustering was also verified with the DAVID functional annotation tool [31], and the PCA function was used to determine the most representative genes for each cluster. The ten most recurrent and unique genes for each GO group were selected and listed in Table 4.

**Table 4:** List of selected transcripts that were significantly modulated in the HK of fish fed the Sel-Plex enriched diet^2^

Trait	SeC	SeP	CP	SeP^5^	Acc. Number^6^	Identity^7^	HGNC	Gene Function^9^
Identifier^1^	HK^2^	HK^3^	HK^4^	CP			Symbol^8^	
TC171135	4.2	−1.6	−1.1	3.0	XM_001922673	Ring finger protein 111	RNF111	Enzyme linked receptor protein signaling pathway
								macromolecule catabolic process
CUST_216_	3.7	−1.1	2.8	1.2	NM_1195534.1	MHC class II beta chain	HLA-DPB1	Cellular response to organic substance
PI429021944								Cellular response to chemical stimulus
TC155958	2.5	−2.4	−1.1	1.2	DQ683253	Protein tyrosine phosphatase alpha	PTPRA	Cellular response to organic substance
								Enzyme linked receptor protein signaling pathway
TC132551	2.2	−1.8	1.2	1.0	NM_001124648	Insulin-like growth factor binding protein 1	IGFBP7	Cellular response to organic substance
								Cellular response to chemical stimulus
TC150601	2.2				AF062496	Insulin receptor a	INSR	Glucose metabolic process
								Hexose metabolic process
TC153429	−2.3	6.5	1.9		NM_001141739	Aspartate aminotransferase cytoplasmic	GOT1	Monosaccharide biosynthetic process
								Gluconeogenesis
TC155657	6.2	−1.2	1.3	4.1	BT072052	Thioredoxin domain-containing protein 5	TXNDC5	Intracellular transport
TC134082	5.0				NM_200685	Nucleoporin 98	NUP98	Nuclear transport
								cellular protein complex assembly
TC167412	3.6	−1.1	1.3	2.5	NM_001177932	SEC24 family member D	SEC24D	Cytoplasmic transport
								Macromolecular complex assembly
TC141904	2.8	2.3	5.2	1.2	NM_001124423	C-x-c chemokine receptor type 3B variant 1	CXCR3	Maintenance of location
TC147797	2.1	1.4	2.0	1.5	NM_201305	Karyopherin alpha 4	KPNA4	Cytoplasmic transport
								Nuclear transport
TC164726	2.0				NM_001025530	SEC62 homolog	SEC62	Intracellular transport
TC146558	3.8	−1.4	1.6	1.7	NM_001141745	Splicing factor 3a subunit 1	SF3A1	mRNA splice site selection
								Spliceosomal complex assembly
TC171125	2.3	1.0	2.5	−1.1	NM_001173829	Tuftelin-interacting protein 11	TFIP11	Spliceosomal complex
								Ribonucleoprotein complex organization
TC147076	2.3	1.2	2.7	−1.0	NM_001139882	Cleavage and polyadenylation specific factor 3	CPSF3	mRNA splicing, via spliceosome
								RNA splicing, via transesterification reactions
TC143716	2.1				BT072055	RNA-binding protein 5	RBM5	Spliceosomal complex assembly
								ribonucleoprotein complex organization
TC142487	2.0	1.3	3.0	−1.1	NM_001173772	Polyadenylate-binding protein 2	PABPN1	mRNA splicing, via spliceosome
								RNA splicing, via transesterification reactions
TC171058	−4.1	−1.0	−3.2	−1.3	NM_001165069	U1 small nuclear ribonucleoprotein C	SNRPC	mRNA splice site selection
TC161955	5.0	−1.9	1.4	1.9	BT059981	Hemoglobin subunit alpha-4	HBA1	Protein complex assembly
								Protein maturation
TC156216	4.9	−1.4	3.5	1.0	NM_214766	Mediator of RNA polymerase II	MED25	Protein/macromolecular complex assembly
TC161655	3.7	−1.5	2.1	1.2	NM_001137665	NCK-associated protein 1	NCKAP1	Protein/macromolecular complex assembly
TC136900	3.6	−1.7	2.2	−1.0	NM_213485	Cullin-associated and neddylation-dissociated 1	CAND1	Protein/macromolecular complex assembly
IMM 315	3.3	−1.8	2.3	−1.3	BT072012	Myeloperoxidase	MPO	Cellular protein complex assembly
								Myofibril
TC135948	3.2	−1.2	−1.1	2.9	NM_001140517	Proteinase-activated receptor 2	F2RL1	Cellular protein complex assembly
								Mitotic cell cycle phase transition
TC138630	10.3	−1.1	1.8	5.2	BT049107	Protease regulatory subunit 4	PSMC1	ATPase activity
								Mitotic cell cycle phase transition
TC159410	9.3	1.2	8.9	1.2	not-annotated	not-annotated	SMARCAD1	Nucleoside-triphosphatase activity
								Pyrophosphatase activity
TC157271	5.2	−2.5	1.5	1.4	XM_001920063	Myosin heavy polypeptide 9b non-muscle	MYH9	ATPase activity
								Hemopoiesis
TC171400	3.5	−1.1	1.3	2.3	NM_001140090	PDZ domain-containing protein 1	KIF1B	ATPase activity
								Nucleoside-triphosphatase activity
TC155666	3.4	−1.3	1.8	1.5	BT050023	Protease regulatory subunit 8	PSMC5	ATPase activity
								Mitotic cell cycle phase transition
TC158128	−4.1				BT060239	Rho-related GTP-binding protein	RHOQ	Nucleoside-triphosphatase activity
								Pyrophosphatase activity
TC169119	3.0				BT045702	Tubulin alpha chain	TUBA1A	Cellular protein complex assembly
								Mitotic cell cycle phase transition
TC163518	2.8	1.0	4.3	−1.5	NM_001140179	Targeting protein for Xklp2	TPX2	Mitotic cell cycle process
TC154893	2.7				CU861476	Adenomatous polyposis coli	APC	Cellular protein complex assembly
								Maintenance of location
TC166756	2.2	−1.2	1.4	1.3	NM_001140263	Lissencephaly-1 homolog B	PAFAH1B1	Cellular component disassembly
								Mitotic cell cycle phase transition
TC163683	2.2	1.5	2.1	1.6	NM_001139958	Cell division cycle 27	CDC27	Mitotic cell cycle phase transition
								Mitotic cell cycle process
TC169614	−2.4	1.7	−2.0	1.4	AB076182	Myosin heavy chain	TUBB	Mitotic cell cycle phase transition
								Cellular protein complex assembly
TC157158	4.2				NM_001140534	Transketolase	TKT	Monosaccharide biosynthetic process
								Glucose metabolic process
TC137888	3.7	1.3	7.8	−1.6	NM_001173578	SMEK homolog 2 suppressor of mek1	SMEK1	Monosaccharide biosynthetic process
								Gluconeogenesis
TC164289	2.4	2.4	6.2	−1.1	AF246148	6-phosphofructo-2-kinase/	PFKFB3	Glucose metabolic process
						fructose-2,6-biphosphatase 9		Hexose metabolic process
TC157094	2.4				BT072470	UDP-N-acetylglucosamine-peptide N-acetylglucosaminyltransferase 110 kDa subunit	OGT	Monosaccharide biosynthetic process
								Glucogenesis
TC150298	−3.6				NM_131108	Type I cytokeratin	KRT17	Monosaccharide biosynthetic process
								Gluconeogenesis
TC156671	−3.7	1.6	−2.9	1.2	NM_001128704	Pyruvate dehydrogenase kinase, isozyme 4	PDK2	Monosaccharide biosynthetic process
TC147626	5.5	−1.4	1.6	2.4	CR382377	B-cell CLL/lymphoma 11Ba	BCL11B	Hemopoiesis
								Cell homeostasis
TC169770	2.6	1.2	3.6	−1.2	NM_001141785	Replication protein A 70 kDa DNA-binding subunit	RPA1	Mitotic cell cycle phase transition
								Cell homeostasis
TC140328	2.2				DQ143177	NOTCH protein-like	NOTCH2	Hemopoiesis
								Notch signaling pathway
IMM 476	2.0	−1.3	1.1	1.4	NM_001160476	Precursor of second macrophage	CSF1	Hemopoiesis
						colony-stimulating factor		Cell homeostasis
TC138601	2.0				NM_001140256	TGF-beta receptor type-2	TGFBR2	Hemopoiesis
								Hematopoietic/lymphoid organ development
IMM 581	−2.2				NM_001185029	Interleukin 17C1	IL17C	Hemopoiesis
TC167544	6.8	1.9	6.8	1.9	AF542091	Low density lipoprotein receptor	LDLR	Cell homeostasis
								Viral process
TC133169	5.6	−2.0	1.1	2.5	FM207660	Partial nIL-1 F gene for novel IL-1 cytokine	ACE2	Viral process
						family member exon 8		Protein maturation
TC136993	4.6	1.3	3.1	1.9	BT048700	Vesicle-associated membrane	VAPB	Viral process
						protein-associated protein B		Symbiosis, through parasitism
TC157378	2.5	−1.5	−1.4	2.4	BT057398	SNARE-associated protein Snapin	SNAPIN	Viral process
								Symbiosis, through parasitism
TC137075	2.3	−1.6	−1.2	1.8	AB208639	AB11 family interacting protein 4 (class II) a	RAB11FIP4	Viral process
								Symbiosis, through parasitism
TC160444	2.3				HQ206612	Structure specific recognition protein 1	SSRP1	Viral process
								Symbiosis, through parasitism

The selection was based on the results of the GO analysis (Fig. 6). Genes with a corresponding microarray feature code^1^ found involved in a biological process significantly altered by the experimental diet in the HK were selected. If the transcripts were significantly modulated also in fish fed the experimental diet enriched with and injected with poly(I:C)^3^, or in the same tissue of fish fed the control diet and injected with poly(I:C)^4^, these values are given. Also the fold change of the expression of the same targets between these last two groups is reported, as given from Genespring software^5^. All the transcripts shown were significantly modulated at *p* < 0.05 following the Benjamini–Hochberg correction and had a fold change ≥2. Accession numbers of the cDNA sequences^6^, their identity^7^ and the corresponding human orthologue^8^ determined by BLASTx within the Ensemble database are reported. For each gene the function assigned by ClueGO software is also indicated. SeC represents the groups comparison addressed to analyse the effects of the Sel-Plex enriched diet. CP and SeP instead represent the comparisons addressed to analyse the effect of poly(I:C) stimulation on fish fed either a control diet or the experimental diet respectively

Induced mRNAs were associated with ATPase activity and cell mitotic processes, such as the proteasome regulatory subunits 4 and 8 (corresponding to human proteasome 26S subunit ATPase 1 (PSMC1) and PSMC5, respectively). Proteasomes are distributed throughout eukaryotic cells at a high concentration and are mainly involved in the ATP-dependent degradation of ubiquitinated proteins in a non-lysosomal pathway. Two other highly induced transcripts matched with human SMARCAD1 (SWI/SNF-related, matrix-associated actin-dependent regulator of chromatin, subfamily a, containing DEAD/H box) and SMARCA4 (SWI/SNF-related, matrix-associated actin-dependent regulator of chromatin, subfamily a, member 4). Members of this family of proteins have helicase and ATPase activities, and they regulate transcription of certain genes by altering the chromatin structure around those genes. Also a transcript with homology to the *D. rerio* myosin heavy polypeptide 9b (MYH9B) gene was highly up-regulated: this gene encodes for a non-muscle myosin that exerts several important functions, including cytokinesis, cell motility and maintenance of cell shape.

Several up-regulated transcripts were involved in the cell cycle. Among these, there were several sequences matching to human genes encoding for proteins involved in microtubule organization and turnover, such as tubulin alpha (TUBA1A), kinesin family member 2C (KIF2C), and TPX2 microtubule-associated (TPX2). A few other transcripts in this category were also broadly involved in the cell cycle, such as cell division cycle 27 (CDC27) and polo-like kinase 1 (PLK1). Up-regulated transcripts for several proteins involved in monosaccharide metabolism, such as insulin receptor (INSR), transkelotase (TKT), 6-phosphofructo-2-kinase/fructose-2,6-biphosphatase 9 (PFKFB3) and ectonucleotide pyrophosphatase/phosphodiesterase 1 (ENPP1) were also found.

Genes involved in hematopoiesis and immune system development were found increased. A transcript matching human B-cell CLL/lymphoma 11B (zinc finger protein) (BCL11B) was highly up-regulated; the function for this protein, although unclear, is believed to be involved in differentiation and survival of lymphocytes. A transcript for a NOTCH protein-like (matching human NOTCH2) was also found up-regulated. Notch family members are highly conserved proteins, which regulate the interaction between physically adjacent cells, by triggering a variety of developmental processes and control cell fate decisions. Also the mRNA for trout colony stimulating factor 1 (CSF1) was induced. This cytokine plays an essential role in the regulation of survival, proliferation and differentiation of hematopoietic precursor cells, especially mononuclear phagocytes, such as macrophages and monocytes. TGF-β receptor type-2 (corresponding to human transforming growth factor, beta receptor II 70/80 kDa, TGFBR2) was induced as well. This is a transmembrane protein that binds TGF-β and phosphorylates proteins, which then enter the nucleus to regulate the transcription of a subset of genes related to cell proliferation.

A number of up-regulated mRNAs were found associated with viral processes and parasite symbiosis. Some of the corresponding proteins for those transcripts are involved in DNA replication and repair (such as structure specific recognition protein, SSRP1), vesicle trafficking (vesicle-associated membrane protein-associated protein B and C (VAPB) and SNARE-associated protein, SNAPIN) and endocytosis (RAB11 family interacting protein 4 (class II), RAB11FIP4), cellular uptake and plasma transport of cholesterol (low density lipoprotein receptor, LDLR). In human studies, some of the genes are found to be associated with intracellular virus entry, replication and final viral burden: such as LDLR that is associated with hepatitis virus entry and replication, and SSRP1 associated with herpes virus [32, 33]. However it is difficult to say if the expression of these genes can have similar implications for viral infection in fish.

### Analysis of the differential transcript response to poly(I:C) treatment in the HK of fish fed a supranutritional level of Sel-Plex

**Fig. 7:**
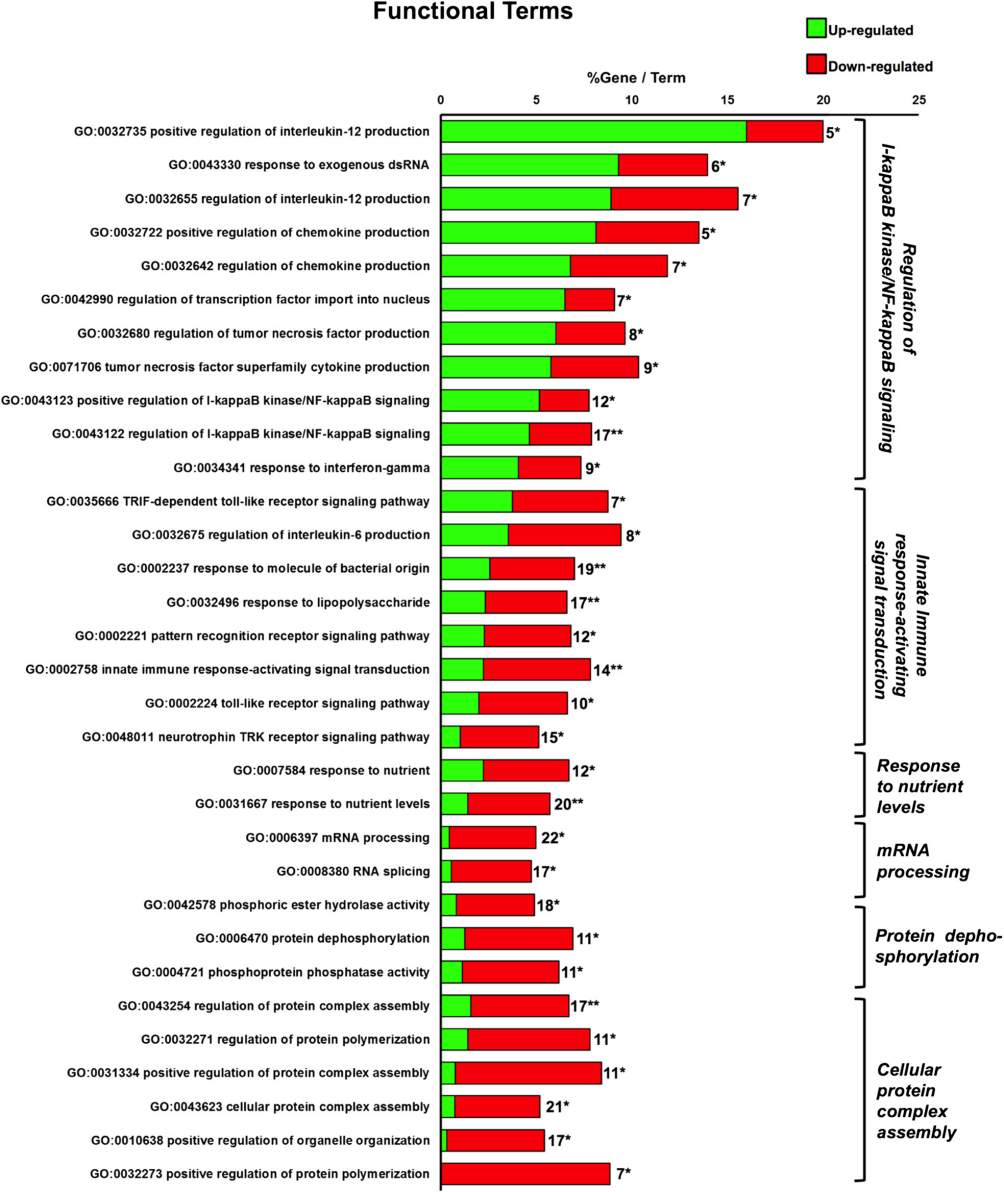
Functional terms enrichment of the identified transcripts in the HK significantly altered by poly(I:C) and 4 mg Se Kg^−1^. The bars represent the percentage of genes found compared to all the genes associated with the term, and the number of genes is displayed. The minimum number of genes assigned for each term was three. A two sided hypergeometric method was applied as statistical test and the *p* values were corrected with the Bonferoni step-down method. Only terms with a *p* < 0.05 are shown. A Kappa score equal to four was used as cut-off for GO term grouping. The level of significance for terms and groups is indicated with an asterisk(s): “*”*p* < 0.05 and “**”*p* < 0.001

In the HK, 344 transcripts were found differentially expressed between the two diet groups, after poly(I:C) stimulation (Fig. 4). Among these, 200 were also significantly modulated by Sel-Plex when compared to individuals injected with vehicle. However, we decided to include all the 344 transcripts that were significantly modulated, albeit to different extents, in the fish stimulated with poly(I:C) on the two different diet regimes, to analyse the interactive effect between Sel-Plex and the response to poly(I:C). This set of transcripts was divided into two clusters: targets that had a higher fold change in the group fed the diet enriched with Sel-Plex versus the control group (for simplicity called “up-regulated”), and targets that had a higher fold change in the group fed the control diet versus the Sel-Plex group (for simplicity called “down-regulated”). From the GO analysis in ClueGO, 138 transcripts contributed to a biological process (Fig. 7). All the significant GO are subdivided under the term considered most relevant and descriptive for the entire group. In Additional file 5: Figure S3, all the GO terms are shown grouped into functional networks and the most statistically significant term for each group is highlighted. In both the representations of the GO analysis, the contribution of the up-regulated and down-regulated clusters of transcripts is visualized.

As shown, some GO term categories (response to nutrient, mRNA processing and cellular protein complex assembly), that were positively regulated when examining the effect of Sel-Plex alone on this tissue, appear to be affected when comparing the two groups injected with poly(I:C). This output might be due to a large set of transcripts that were up-regulated when compared to the two diet groups injected with vehicle. However, they were down-regulated in the contrast between the experimental diet group given poly(I:C) versus the same diet group injected with PBS. Therefore, attention was focused on the main GO network produced from this analysis, exclusively to examine the effect of Sel-Plex on fish antiviral responses. Two interconnected GO groups arose from this analysis: regulation of I-kappaB kinase/NF-kappa B (NF-κB) signalling mostly due to transcripts more highly regulated in the group fed Sel-Plex, and the innate immune response-activating signal transduction, which instead resulted from a higher expression of certain genes in the control diet group.

Within these two GO groups, IL-12 and chemokine production together with the type-II IFN response were enhanced by the Sel-Plex supplementation, whereas production of IL-6, the bactericidal response and toll-like receptor downstream signalling were slightly (but significantly) reduced in the same group. Table 5, lists the genes that were grouped within these terms that are reported but were not significantly altered in the HK of fish fed the experimental diet and injected with PBS.

**Table 5:** List of selected transcripts significantly modulated in the HK of fish injected with poly(I:C) and dependant on prior feeding regime. The selection was based on the results of the GO analysis (Fig. 7)

Trait	SeC	SeP	CP	SeP^5^	Acc. Number^6^	Identity^7^	HGNC	Gene Function^9^
Identifier^1^	HK^2^	HK^3^	HK^4^	CP			symbol^8^	
CUST_201_	−1.2	19.2	5.9	2.7	FM864346.1	Interferon gamma2	IFNG	Positive regulation of interleukin-12 production
PI429021944								
IMM 478	−1.1	15.1	8.0	1.7	NM_001124446	Glycogen synthase kinase binding protein	GBP1	Response to interferon-gamma
IMM 851	1.0	13.5	6.8	2.0	AF401631	TNF decoy receptor	TNFRSF6B	Regulation of cellular response to stress
TC151161	−1.1	9.4	4.9	1.7	BT072291	Salmo salar clone ssal-rgf-519–364,	MAVS	Positive regulation of chemokine production
						unknown large open reading frame		Response to exogenous dsRNA
TC160031	−1.3	7.6	2.8	2.1	BT058838	Metalloproteinase inhibitor 3	TIMP3	Response to organic substance
TC132324	−1.1	5.6	3.3	1.5	NM_001124412	Toll-like receptor 22	TLR3	Positive regulation of interleukin-12 production
								Positive regulation of chemokine production
TC144460	−1.2	5.5	2.7	1.7	BT074153	Sorcin	SRI	Regulation of transcription factor import into nucleus
								Cellular transition metal ion homeostasis
TC138806	−2.5	5.4	2.0	1.1	EU481821	Salmo salar physical map contig 483 genomic sequence	NR2F2	Positive regulation of macromolecule metabolic process
								Regulation of DNA binding
TC142451	−1.5	4.5	1.8	1.6	BT048169	Lipopolysaccharide-induced tumor necrosis factor-alpha factor	LITAF	Regulation of transcription factor import into nucleus
								Regulation of NF-kappaB import into nucleus
TC144371	−1.3	4.1	2.4	1.3	NM_001124746	Transducer/activator of transcription	STAT1	Response to exogenous dsRNA
								Response to interferon-gamma
TC155255	−1.4	3.9	1.3	2.2	BT072728	B-cell lymphoma 3-encoded protein	BCL3	Regulation of transcription factor import into nucleus
								Regulation of NF-kappaB import into nucleus
TC155255	−1.4	3.9	1.3	2.2	BT072728	B-cell lymphoma 3-encoded protein	BCL3	Regulation of transcription factor import into nucleus
								Regulation of NF-kappaB import into nucleus
TC148036	−1.2	3.4	2.5	1.2	NM_001165385	Macrosialin	SSC5D	Response to molecule of bacterial origin
TC164429	−1.2	2.4	1.4	1.5	FR751081	Caspase 3	CASP3	Neurotrophin TRK receptor signaling pathway
CUST_118_	−1.1	2.4	1.5	1.5	NM_001124438	Interferon regulatory factor 2	IRF2	Response to interferon-gamma
_PI420312184								
TC162287	−1.2	2.4	1.7	1.2	not-annotated	not-annotated	RIPK2	Positive regulation of interleukin-12 production
								Positive regulation of chemokine production
TC161320	−1.2	2.2	1.5	1.2	BT057463	Proteasome subunit alpha type-6 putative	PSMA6	Regulation of G1/S transition of mitotic cell cycle
CUST_75	−1.0	2.1	1.6	1.3	GQ169787.1	TNF receptor superfamily member 5A	CD40	Positive regulation of interleukin-12 production
_PI429021944								
TC137335	−1.5	−1.2	−2.2	1.2	not-annotated	not-annotated	ECT2	Maintenance of protein location in cell
								Positive regulation of I-kappaB kinase/NF-kappaB signaling
TC141297	−1.6	−1.2	−2.4	1.2	BT073959	Proteasome subunit beta type 1-A	PSMB1	Regulation of G1/S transition of mitotic cell cycle
								Antigen processing and presentation of peptide antigen
TC144499	−1.2	−1.4	−2.2	1.3	NM_001165391	Cyclin D1	CCND1	Regulation of G1/S transition of mitotic cell cycle
								response to nutrient
TC134399	−1.3	−1.5	−2.1	1.1	BT072801	Mitogen-activated protein kinase 7-interacting protein homolog 2	TAB2	Toll-like receptor signaling pathway
								MyD88-dependent toll-like receptor signaling pathway
TC145638	−1.4	−1.5	−2.4	1.1	BT027690	CGX87-B08	TTK	Positive regulation of signal transduction
								Positive regulation of cell proliferation
TC142766	−1.4	−1.5	−2.4	1.1	FP236858	Zebrafish DNA sequence from clone CH73-339E3	TJP1	Response to molecule of bacterial origin
								Response to lipopolysaccharide
TC168733	−1.5	−1.5	−2.5	1.1	BT059118	Choline transporter-like protein 2	SLC44A2	Regulation of I-kappaB kinase/NF-kappaB signaling
								Positive regulation of I-kappaB kinase/NF-kappaB signaling
TC159957	−1.7	−1.7	−3.1	1.1	BT072217	Fibronectin	FN1	Defence response
								Response to wounding
TC132905	3.0	1.5	3.4	1.3	NM_001124288	Hypoxia-inducible factor 1 alpha subunit	HIF1A	Regulation of chemokine production
								positive regulation of chemokine production
TC156038	1.5	1.5	2.5	−1.2	BT044876	Ras-related C3 botulinum toxin substrate 1 precursor	RAC1	Positive regulation of protein polymerization
TC169458	1.9	1.8	2.5	1.4	BT074060	NF-kappa-B inhibitor alpha	NFKBIA	Regulation of NF-kappaB import into nucleus
								Response to exogenous dsRNA
TC135280	1.5	1.6	2.4	1.0	BT044949	Tyrosine-protein kinase HCK	HCK	Positive regulation of protein polymerization
								Regulation of actin filament polymerization
TC151093	1.2	1.5	2.3	−1.2	BT044981	Histone deacetylase 3	HDAC3	Neurotrophin TRK receptor signaling pathway
TC137681	1.7	1.3	2.3	−1.1	BT045910	Mitogen-activated protein kinase-activated	MAPKA	Regulation of tumor necrosis factor production
								Regulation of interleukin-6 production
TC148701	1.7	−1.0	2.1	−1.2	NM_001171899	Red cell arrestin 2	ARRB2	Regulation of interleukin-12 production
								Regulation of tumor necrosis factor production
TC135368	1.4	1.0	2.1	−1.4	NM_001172498	CCAAT/enhancer binding protein beta2	CEBPB	Regulation of interleukin-6 production
IMM 136	1.8	1.1	2.0	−1.0	AJ620466	Complement receptor-like protein 1 splice variant 2	CD55	Response to molecule of bacterial origin
								Response to lipopolysaccharide
IMM 505	1.6	1.1	2.0	−1.1	DQ399551	Dual specificity phosphatase 6 (dusp6)	DUSP6	Toll-like receptor signaling pathway
								MyD88-dependent toll-like receptor signaling pathway
TC167434	1.8	1.1	2.0	1.0	NM_001140341	Sequestosome-1	SQSTM1	regulation of I-kappaB kinase/NF-kappaB signaling
								neurotrophin TRK receptor signaling pathway
TC132407	1.5	1.2	2.0	−1.1	NM_001124559	cAMP-dependent transcription factor	ATF1	toll-like receptor signaling pathway
								MyD88-dependent toll-like receptor signaling pathway
IMM 223	1.5	−2.0	−1.2	−1.1	BT047880	Heme oxygenase putative mRNA	HMOX1	regulation of chemokine production
								cellular transition metal ion homeostasis

Genes with corresponding microarray feature code^1^ found involved in a biological process significantly altered by the experimental diet in the HK were selected. If the transcripts were significantly modulated also in fish fed the experimental diet enriched with 4 mg Se Kg^−1^ and injected with poly(I:C)^3^, or in the same tissue of fish fed the control diet and injected with poly(I:C)^4^, these values are given. Also the fold change of the expression of the same targets between these last two groups is reported, as given from Genespring software^5^. All the transcripts shown were significantly modulated at *p* < 0.05 following the Benjamini–Hochberg correction and had a fold change ≥2. Accession numbers of the cDNA sequences^6^, their identity^7^ and the corresponding human orthologue^8^ determined by BLASTx within the Ensemble database are reported. For each gene the function assigned by ClueGO software is also indicated. SeC represents the groups comparison addressed to analyse the effects of the 4 mg Se Kg^−1^ diet. CP and SeP instead represent the comparisons addressed to analyse the effect of poly(I:C) stimulation on fish fed either a control diet or the experimental diet respectively

As also shown from the *q*PCR results, the transcript for trout IFN-γ2 was significantly up-regulated by Sel-Plex supplementation. Other mediators involved in antiviral responses were induced more in the supplemented group, such as: toll-like receptor 22 (TLR22), signal transducer/activator of transcription 1 (STAT1), glycogen synthase kinase binding protein (GBP1) and mitochondrial antiviral signalling protein (MAVS). A trout transcript for tumor necrosis factor (TNF) decoy receptor (corresponding to human tumor necrosis factor receptor superfamily, member 6b, decoy, TNFRSF6B) was also up-regulated upon Sel-Plex supplementation; this protein belongs to the TNF receptor superfamily and acts as a decoy receptor that neutralizes FAS ligand action, protecting cells from apoptosis. A lipopolysaccharide-induced TNF factor mRNA (LITAF) and a transcript for microsialin (CD68) were also highly induced in the supplemented group. LITAF is a transmembrane glycoprotein expressed by monocytes and tissue macrophages that plays a role in phagocytic activities of tissue macrophages (both in intracellular lysosomal metabolism and extracellular cell-cell and cell-pathogen interactions), whereas CD68 is a potent stimulator of macrophages that induces secretion of TNF-alpha and other inflammatory mediators, possibly leading to p53-induced apoptosis. Also the induction of transcripts for signal transduction mediators, such as caspase 3 (CASP3) and IFN regulatory factor 2 (IRF2) were enhanced to some degree in the group fed Sel-Plex enriched diet. IRF2 is known to compete with IRF1 to inhibit the expression of type I IFNs. However transcripts for trout type I IFN were equally induced by poly(I:C) in the two diet groups fed the control and the Sel-Plex enriched diet, as shown by both *q*PCR and microarray platforms. A transcript matching to the human receptor-interacting serine-threonine kinase 2 (RIPK2) was also more highly induced upon Sel-Plex supplementation; this protein is a positive regulator of NF-κB and its expression might be correlated with the reduced expression of NF-κB inhibitor alpha (matching to human nuclear factor of kappa light polypeptide gene enhancer in B-cells inhibitor alpha, NFKBIA) in the same experimental group.

Some transcripts for proteins involved in the pro-inflammatory response and acute phase response were more up-regulated in the group injected with poly(I:C) and fed a control diet, compared to the group fed Sel-Plex: as seen with Tyrosine-protein kinase (HCK), mitogen-activated protein kinase-activated protein kinase 2 (MAPKAPK2) and CCAAT/enhancer binding protein beta2 (CEBPB).

Finally the most responsive transcripts modulated by Sel-Plex upon poly(I:C) stimulation are reported. The two groups injected with poly(I:C) on the different diet regimes were compared in Genespring and the 20 targets that were most up- and down-regulated, and that were not already mentioned above were listed (Additional file 6: Table S3).

One of most responsive targets in this comparison was a transcript for an uncharacterized protein that from the BLAST analysis matched human lactalbumin alpha (LALBA), a lysozyme–like protein that binds calcium and zinc, with a possible antibacterial and antitumor activity. Also transcripts for fish orthologues of human Paf1/RNA polymerase II complex component (CTR9), protein tyrosine phosphatase receptor type F (PTPRF) and zinc finger FYVE domain containing 9 (ZFYVE9) were more highly induced in the group fed the Sel-Plex enriched diet. CTR9 is a component of the PAF1 complex (PAF1C) which has multiple functions during transcription by RNA polymerase II and it is implicated in the regulation of development and maintenance of embryonic stem cell pluripotency. PTPRF possesses an intrinsic protein tyrosine phosphatase activity that regulates a variety of cellular processes including cell growth, differentiation, mitosis, and oncogenic transformation. ZFYVE9 is a double zinc finger motif-containing protein that participates in the transforming growth factor-beta (TGF-*β*) signalling pathway. A sequence corresponding to the mRNA for human Runt-Related Transcription Factor 1 (RUNX1) was also up-regulated. This protein is a heterodimeric transcription factor that binds to the core element of many enhancers and promoters and is thought to be involved mainly in the development of normal hematopoiesis. Another transcript differentially expressed was the trout aryl hydrocarbon receptor nuclear translocator (ARNT) which plays an important role in xenobiotic responses, and after binding to a ligand it translocates into the nucleolus where it induces the expression of factors involved in xenobiotic metabolism. It can also form a heterodimer with HIF1A and contributes to the response to hypoxia. Finally, chemokine (C-X-C Motif) ligand 11 (CXCL11) expression was doubled in the group fed the Sel-Plex enriched diet. This chemokine is induced by IFN-γ, is the dominant ligand of the CXCR3 receptor (see above) and has an important function to trigger the chemotactic response of T-cells.

Of those genes negatively impacted by Se supplementation, a transcript for ATPase Na^+^/K^+^ transporting beta 4 polypeptide (ATP1B4) was detected, a protein that in placental mammals interacts with the nuclear transcriptional co-regulator SKIP and may be involved in the regulation of TGF-*β* signalling. Two transcripts for C-terminal binding protein 1 (CTBP1) and programmed cell death 6 (PDCD6) were also down-regulated in this comparison. The first is a phosphoprotein that acts as a transcriptional repressor and plays a role in cellular proliferation. The second is a calcium binding protein that participates in T cell receptor-, Fas, and glucocorticoid-induced programmed cell death. Finally an mRNA for C-type lectin domain family 17 member A (CLEC17A) was down-regulated and is a cell surface receptor which may be involved in carbohydrate-mediated communication between cells in germinal centres. These sites are missing in fish, but melano-macrophage centres (MMCs) are thought to have a similar function and to represent primitive germinal centres [34].

### Selenoprotein transcript responses

A few probes for trout selenoproteins were significantly modulated on the array (Additional file 7: Table S5), however in this specific study Sel-Plex supplementation did not alter the expression of these probes. This was not surprising, since only a small induction was seen previously for several of these target genes on a more sensitive platform (*q*PCR). With the exception of the mRNAs for trout glutathione peroxidase 2 (the BLAST result being most similar to trout GPx1b1, [25]) and cytosolic thioredoxin reductase 1 (the BLAST result being most similar to trout TrxR3a, [26]), which were up-regulated upon poly(I:C) stimulation, all selenoprotein transcripts were down-regulated, especially the ones corresponding to trout deiodinase (DIOs).

## Discussion

Se is a fundamental dietary element that plays an important role in organism homeostasis. As an essential component of several selenoproteins with reductive capacity (primarily GPxs and TrxRs), Se plays an important role in controlling the redox milieu in an organism. Moreover, Se is required for metabolism of thyroid hormone, being fundamental for the proper functioning of DIO proteins. SelP is another important component of this protein family; it is well known as the main selenoprotein responsible for organism Se homeostasis and transport, however a role in the immune response, especially in inflammation, has also been proposed for this protein [35, 36]. The role of Se within the immune system has been investigated extensively [13] . It is well established that a low Se status can compromise responses to stress and immune challenges within an organism [37–39] . Se supplementation might either counteract compromised physiological status due to Se deficiency, or ameliorate the basal immune function and represent a way to help recovery from certain pathological conditions [40–42] . Several immune-related processes rely on Se and selenoprotein function, however the molecular mechanisms behind the interaction of this element with immune system functions are not fully understood [14].

In fish aquaculture, pathogens, especially viruses, represent a constant threat to production. The mode of action of many fish viruses during infection is still under investigation, and there is a continuous effort to find possible solutions to enhance fish natural defences. Functional feeds might represent an alternative to improve fish natural defences. They are particular diet compositions, containing substitutes or supplements with the aim to improve fish fitness and immune defences. Elements such as Se, provided at supranutitional levels and below toxic concentrations, might represent a natural and sustainable solution to improve fish health. In our previous study [20] we proposed that fish, salmonids in particular, may require higher levels of Se compared to mammals and more than current legislation allows (0.5 mg Kg^−1^ dry mass). We showed that Se delivered as Sel-Plex to reach supplementation levels between ~5–9 mg Se Kg^−1^, is well absorbed and might improve selenoprotein synthesis. These findings are in line with other studies that found that salmonids might benefit from Se at this range of concentrations, especially if delivered in organic form and when fish are under stressful conditions.

In this study, we investigated the potential effect of Sel-Plex supplementation on fish antiviral defences, induced by poly(I:C) injection. To this end, we carried out a transcriptomic analysis by microarray on both liver and HK from rainbow trout challenged with this viral mimic after being fed a diet enriched with Sel-Plex. After a first *q*PCR screening a slight but significant induction of selenoprotein transcripts was seen in the liver of the group supplemented with 4 mg Se Kg^−1^. Interestingly in the same experimental group a significant increase of type-II IFN (especially IFN-γ2) was also detected, with downstream molecules such as CXCL11 and viperin following the same trend. However, with increasing concentration of Sel-Plex, there was also a small decrease in type-I IFN (mainly IFN-b) and selected cellular receptors involved in viral sensing, such as TLR9, LGP2 and MDA5 (the latter significantly). The preliminary *q*PCR results led to the selection of the group fed 4 mg Kg^−1^ Sel-Plex for the microarray analysis together with the control group. The entire trout array was re-annotated to the human genome, therefore for each fish gene the human orthologue was found by BLAST analysis in the Ensemble database. Human and fish have quite different genome structures, mainly due to the whole genome duplications that have occurred in the teleost lineage. Several genes present as a single copy in humans can be present in fish as two or more (especially in the case of salmonids) isoforms. Some of these multiple copies are pseudogenes but in other cases these multiple isoforms can exert different biological functions, increasing the complexity of various pathways. Hence, in taking this approach there was a risk that only partial information would be retrieved from the transcriptomic analysis, but as the human genome remains the most annotated, it gave the possibility to carry out a more extensive analysis. It also allowed use of more bioinformatic tools, giving the possibility of a more flexible analytical approach. For example, for the GO analysis, ClueGO allowed the up-regulated and down-regulated transcripts to be analysed together and to determine the contribution of the two clusters in the biological pathways found altered in each of the contrasts considered for this study.

It was not possible to perform a network analysis for the processes modulated in liver, mainly due to the small set of mRNAs found altered by the treatment. Overall the response of this organ to the diet was relatively mild. Only a small set of transcripts (20 targets) was processed by the GO analysis, and the main terms were involved in lipid and steroid metabolism. The possible effects of Se supplementation on fish metabolism of lipids, especially steroid and fatty acids, might be an intriguing aspect to investigate further. Should Se be able to increase the fillet nutritional value, for example by improving the content of omega-3 fatty acids, it could have an important impact on human nutrition and fish food marketing. Lipid metabolism can also influence the fish immune response. Recent findings have shown that cholesterol, specifically oxidized derivates such as 25-hydroxycholesterol (25HC), are important mediators during the innate response to pathogens. During virus infection, TLR-mediated signalling in macrophages triggers the induction of an IFN response, which promotes the production of 25HC mediated by STAT1; in turn this oxysterol can act on multiple levels as a potent paracrine inhibitor of viral infection for a broad range of viruses [43]. The present results suggest that Sel-Plex supplementation is able to modulate liver lipid metabolism and increase cholesterol production, possibly enhancing the innate immune response against viral infections. The results also revealed a considerable induction of LDLR in the HK, which might be associated with the pathway just described.

In the liver, changes in the expression of a number of mRNAs for genes involved in sarcomere organization were detected, which generally are expected to be expressed in skeletal and heart muscle exclusively. There is strong evidence in mammalian models for the presence of resident stem cells in the liver and the role of this tissue as a hematopoietic centre [44, 45]. In this regard, a few studies have shown it is possible to differentiate murine liver stem cells into cardiomyocytes [46, 47]. Also a metabolic crosstalk between heart and liver has been described, that plays a pivotal role in the development of hypertrophic cardiomyopathy when a dysregulation of liver lipid metabolism occurs [48]. However, it is difficult to make inferences from such a small set of genes, as to whether there is a real and strong correlation between Se supplementation, lipid metabolism and muscle tissue homeostasis, especially when the process under examination has not been completely explored in a more established animal model and is still totally unknown in fish. The possible influence of Se supplementation on fish fillet quality and composition certainly remain an interesting aspect for further investigation.

Sel-Plex had a greater effect on the HK transcriptomic response. In this tissue a large number of target genes were consistently modulated, most of them positively induced. Terms such as regulation of transcription, mRNA metabolism and protein assembly were found from the GO analysis. However, these are generic terms that link to several biological processes. What was particularly interesting was the positive regulation of the cellular response to nutrients, glucose metabolism, cell cycle processes and hematopoiesis. All the processes mentioned may lead to an improvement of turnover, especially of cells involved in immune responses, which take place in the HK, the hematopoietic centre and main immune tissue in fish. A recent study in zebrafish showed that glucose level can impact the onset and magnitude of hematopoietic stem cell (HSC) production *in vivo*. Specifically, glucose level could enhance RUNX1 gene expression and hematopoietic cluster formation, to finally elicit a dose-dependent effect on embryonic HSCs, through mitochondrial-derived ROS–mediated stimulation of the HIF1Α gene [49]. In this regard, *runx1* and *hif1a* transcripts, together with several other mRNAs for proteins involved in tissue hematopoiesis, appeared up-regulated by the Sel-Plex enriched diet.

In the same tissue, a few transcripts were found that in the GO analysis are associated with processes linked to viral replication. From this finding alone, it is not possible to make any firm conclusions, since the terms associated to these genes are based on studies in mice, with virus specific infection mechanisms in rodent models and humans. This highlights another problem that can occur by using the human genome annotation, however the advantages still compensate for the possible limitations of this approach.

After poly(I:C) injection, transcripts for proteins involved in the antiviral response were found up-regulated upon Sel-Plex supplementation. The mRNA for TLR22, STAT1, IFN-γ2, GPB1 and MAVS were all significantly increased after Se supplementation. Downstream of the IFN-γ pathway, the mRNAs for CXCL11 and its receptor CXCR3 were positively modulated by the experimental diet. All the transcripts found positively regulated by the poly(I:C) challenge in the group fed Sel-Plex clustered into three main GO processes: NF-κB regulation, IL-12 production and the IFN-γ response. These three processes are tightly connected. IFN-γ production is controlled by cytokines secreted from antigen presenting cells (APCs), most notably IL-12 and IL-18; these cytokines serve as a bridge to link infection with IFN-γ production in the innate immune response. Macrophage recognition of many pathogens induces secretion of IL-12 and chemokines, which in turn attract natural killer (NK) cells to the site of inflammation, and IL-12 promotes IFN-γ synthesis in these cells. In macrophages, NK and T-cells, the combination of IL-12 and IL-18 stimulation further increases IFN-γ production, and favours Th1 cell differentiation [50]. NF-κB, as a key transcription factor for a wide variety of genes that control immune responses, is likely playing a central role in cytokine expression and in the propagation of the response during viral challenge [51–53].

In mice models, it has been shown that Se at supranutritional levels (0.5–1 mg Kg^−1^ as Na_2_SeO_3_) during an immune challenge increases production of IL-2 and the expression of the IL-2 receptor α subunit. Consequently the T helper Th1/Th2 balance skews towards a Th1 response, leading to enhanced differentiation/activation of CD4^+^ T-cells and production of IFN-γ [54]. In the same study, it was shown that free thiols, mainly thioredoxin (Trx), are the intracellular mediators responsible for the increased production of IL-2; TrxR, being responsible for the regeneration of the oxidised Trx, becomes a central component in controlling the process. Interestingly, from our preliminary *q*PCR screening an induction of IFN-γ and downstream mediators was seen, but at much higher concentrations (4 mg Kg^−1^) than those considered for supplementation in mammalian models (0.5–1 mg Kg^−1^).

From the GO analysis, it was seen that Sel-Plex supplementation affected TLR signalling and responses to bacteria. Whilst the expression of some of the components of the cell pathogen recognition machinery was reduced, a positive regulation of many immune effectors in the Sel-Plex supplemented group was found when the antiviral defences were stimulated. This suggests other cell recognition components are involved in the mediation of the cellular response to poly(I:C).

The selenoprotein transcripts did not show a strong modulation after Sel-Plex supplementation. Previous studies reported that selenoprotein mRNA may not be suitable in detecting the effects of Se supplementation, especially if using a less sensitive platform such as a microarray [55, 56]. Nevertheless, most of the selenoprotein mRNAs detected (mainly DIOs and SelPs) were down-regulated upon poly(I:C) stimulation; only TrxR mRNA was up-regulated, further confirming the important contribution of this selenoprotein during the organism’s immune response.

## Conclusion

Sel-Plex supplementation had a major impact on the trout HK. Even at the relatively high enrichment level of 4 mg Se Kg^−1^ (dry mass), the additive improved the expression of several genes involved in pathways connected with hematopoiesis and immune system development. In contrast, less of a transcriptomic response was seen in the liver, and the main process modulated by Sel-Plex was related to lipid metabolism. Lipids, especially sterol lipids (e.g. cholesterol and glucocorticoids) and fatty acids (e.g. omega-3 and omega-6 fatty acids), have a major involvement in the immune response and the final quality of the fish fillet, and therefore it is worth investigating further the possible impact of Se supplementation on these processes in fish. During viral PAMP stimulation, Sel-Plex supplementation substantially increased the expression of mediators of antiviral defence. IFN-γ was one of the principal markers for this pathway and interestingly a similar response to Se supplementation has been seen in mammals, albeit at lower inclusion levels of Se than the levels used in this study. These findings, together with past reports in the literature, further support the conclusion that fish might require a higher level of Se than mammals, and an increase over the limit of 0.5 mg Kg^−1^ imposed by current EC legislation should be considered for supplementation in aquaculture. Sel-Plex, being a highly bioavailable supplement of organic Se, might represent the most suitable option for supplementation of fish feeds, to achieve the final aim of improving fish fitness and resistance against immune challenges.

## Availability of supporting data

The experimental hybridisations are available at European Bioinformatics Institute archived under accession number E-MEXP: E-MTAB-2982.

## Additional files

Additional file 1: Figure S1. Diagram outlining the experimental setup and hybridization strategy for microarray analysis. For each experimental group five pools of HK or liver RNA extracted from three individuals were used. For the microarray experiment, the effect of the 4 mg Se Kg^−1^ diet (SeC), plus the effect of poly(I:C) stimulation on fish fed either a control diet (CP) or the 4 mg Se Kg^−1^ enriched diet (SeP) was investigated, in both liver and head kidney. One-way ANOVA was used to compare differences among groups, whilist two-way ANOVA analysis was applied to determine differential responses to poly(I:C) between the two diet groups. The pooled control was an equal mixture of RNA pools from each group. All hybridizations were between experimental groups and the pooled control. (PDF 317 kb) Additional file 2: Figure S2. Principal component analysis of the expression patterns of the four distinct conditions analyzed. Clustering of diets (control diet and Sel-Plex supplementation) and treatments (PBS or poly(I:C)) groups based on the three (left) and two principal components (right), in HK (A) and liver (B). The colours and shapes of the data points indicate diet group and treatment. The x-, y- and z-axes represent PC1, PC2 and PC3, respectively. (PDF 753 kb) Additional file 3: Table S1. Vitamin and mineral composition in the raw material for all diets used in this study. (PDF 61 kb) Additional file 4: Table S2. Real time *q*PCR analysis of genes selected for confirmation of microarray data. Candidate transcripts expression in HK (A) and liver (B) RNA isolated from rainbow trout fed a control diet (CTRL) or a diet enriched with 4 mg Se Kg^**−**1^ (Sel-Plex), and injected with either PBS or poly(I:C). The expression of gene transcripts was quantified by *q*PCR and normalized against the geometric mean of three housekeeping genes (*ef-1α*, *drpII*, *hprt*) from the same samples, and then used for statistical analysis. The transcript expression is reported as a fold change, calculated as the average expression level of stimulated samples divided by that of the controls. The results represent the mean ± SEM from five pools of HK or liver RNA extracted from 3 individuals from 3 different tanks. The letters above the columns indicate values that are statistically significant vs the controls (*p* < 0.05) in the group injected with poly(I:C), with different letters indicating significant differences between the two diet regimes. Asterisks indicate values that are statistically significant vs the control (*p* < 0.05) in the group fed 4 mg Se Kg^**−**1^ and injected with PBS. The primers used are listed in Table 2. (PDF 173 kb) Additional file 5: Figure S3. Grouped annotation network (A) and cluster distribution network (B) of the functional terms listed in Fig. 7 visualised using ClueGO. In both layouts, terms are connected depending on the similarity of their associated genes. The degree of connectivity between terms (edges) is calculated using kappa statistics and the network is shown using the Organic layout algorithm supported by Cytoscape. The size of the nodes reflects the statistical significance of the terms, whereas the thickness of the edge reflects the grade of functional connection between terms. The group leading term is the most significant term of the group. In the grouped annotation network, functional terms are grouped by different colours, each identifying the most significant term of the group. In the cluster distribution network, green and red label functional terms are up- and down-regulated genes, respectively. The colour gradient shows the gene proportion of each cluster associated with the term. (PDF 1254 kb) Additional file 6: Table S3. List of transcripts showing the highest change in the HK of fish injected with poly(I:C) and dependent on prior feeding regime. The selection was based on the results of the GO analysis (Fig. 7). All the genes with corresponding microarray feature code^1^ found involved in a biological process significantly altered by the experimental diet in the HK were selected. If the transcripts were significantly modulated also in fish fed the experimental diet enriched with 4 mg Se Kg^−1^ by adding Sel-Plex and injected with poly(I:C)^3^, or in the same tissue of fish fed the control diet and injected with poly(I:C)^4^ , these values are given. Also the fold change of the expression of the same targets between these last two groups is reported, as given from Genespring software^5^. All the transcripts shown were significantly modulated at *p <* 0.05 following the Benjamini-Hochberg correction and had a fold change ≥2. Accession numbers of the cDNA sequence^6^, their identity^7^ and the corresponding human orthologue^8^ determined by BLASTx within the Ensemble database are reported. For each gene the function assigned by ClueGO software is also indicated. SeC represents the groups comparison addressed to analyse the effects of the diet enriched with 4 mg Se Kg^−1^. CP and SeP instead represent the comparisons addressed to analyse the effect of poly(I:C) stimulation on fish fed either a control diet or the experimental diet respectively. (PDF 145 kb) Additional file 7: Table S5. List of selenoprotein transcripts significantly modulated. All the selenoprotein transcripts modulated in the HK and liver of fish fed the experimental diet enriched with 2 g Kg^−1^ Sel-Plex and injected with PBS, of fish injected with poly(I:C) and fed the experimental diet enriched with 2 g Kg^−1^ Sel-Plex and the control diet respectively, are given. All the transcripts shown were significantly modulated (*p* < 0.05) following the Benjamini–Hochberg correction and had a fold change ≥2. Accession numbers of the cDNA sequences^6^, their identity^7^ and the corresponding human orthologue^8^ determined by BLASTx and BLASTn are reported. SeC represents the groups comparison addressed to analyse the effects of the 2 g Kg^−1^ Sel-Plex diet. CP and SeP instead represent the comparisons addressed to analyse the effect of poly(I:C) stimulation on fish fed either a control diet or the 2 g Kg^−1^ Sel-Plex diet respectively. (PDF 111 kb)
