# Supplementary material for: Impact of selenium supplementation on fish antiviral responses: a whole transcriptomic analysis in rainbow trout (Oncorhynchus mykiss) fed supranutritional levels of Sel-Plex®
Source: BMC Genomics. 2016 Feb 16;17:116. doi: 10.1186/s12864-016-2418-7 (PMC4754912; doi:10.1186/s12864-016-2418-7)

Additional File 1: Figure S1. Diagram outlining the experimental setup and hybridization strategy for microarray analysis. For each experimental group five pools of HK or liver RNA extracted from three individuals were used. For the microarray experiment, the effect of the 4 mg Se Kg^{-1} diet (SeC), plus the effect of poly(I:C) stimulation on fish fed either a control diet (CP) or the 4 mg Se Kg^{-1} enriched diet (SeP) was investigated, in both liver and head kidney. One-way ANOVA was used to compare differences among groups, whilist two-way ANOVA analysis was applied to determine differential responses to poly(I:C) between the two diet groups. The pooled control was an equal mixture of RNA pools from each group. All hybridizations were between experimental groups and the pooled control.

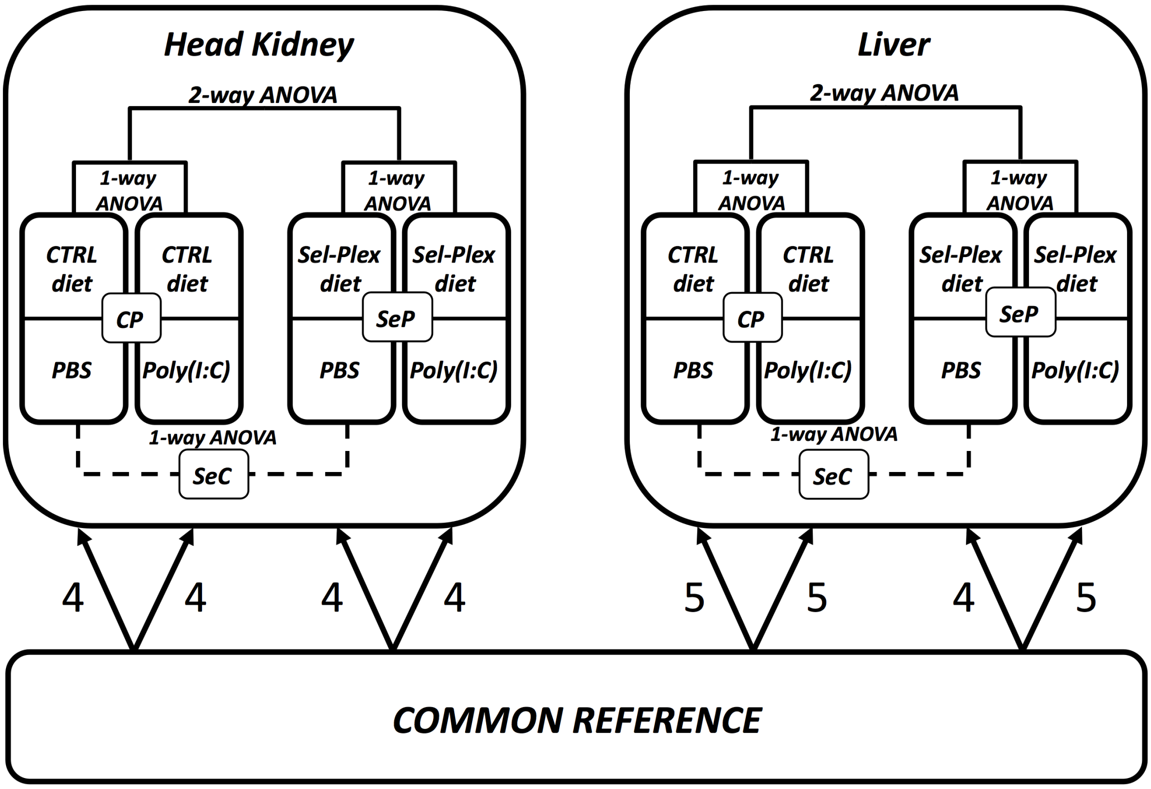

Supplement: Additional file 1: Figure S1. — Diagram outlining the experimental setup and hybridization strategy for microarray analysis. For each experimental group five pools of HK or liver RNA extracted from three individuals were used. For the microarray experiment, the effect of the 4 mg Se Kg−1 diet (SeC), plus the effect of poly(I:C) stimulation on fish fed either a control diet (CP) or the 4 mg Se Kg−1 enriched diet (SeP) was investigated, in both liver and head kidney. One-way ANOVA was used to compare differences among groups, whilist two-way ANOVA analysis was applied to determine differential responses to poly(I:C) between the two diet groups. The pooled control was an equal mixture of RNA pools from each group. All hybridizations were between experimental groups and the pooled control. (PDF 317 kb) [file 12864_2016_2418_MOESM1_ESM.pdf]
