# Supplementary material for: Impact of selenium supplementation on fish antiviral responses: a whole transcriptomic analysis in rainbow trout (Oncorhynchus mykiss) fed supranutritional levels of Sel-Plex®
Source: BMC Genomics. 2016 Feb 16;17:116. doi: 10.1186/s12864-016-2418-7 (PMC4754912; doi:10.1186/s12864-016-2418-7)

Additional File 2: Figure S2. Principal component analysis of the expression patterns of the four distinct conditions analyzed. Clustering of diets (control diet and Sel-Plex supplementation) and treatments (PBS or poly(I:C)) groups based on the three (left) and two principal components (right), in HK (A) and liver (B). The colors and shapes of the data points indicate diet group and treatment. The x-, y- and z-axes represent PC1, PC2 and PC3, respectively.

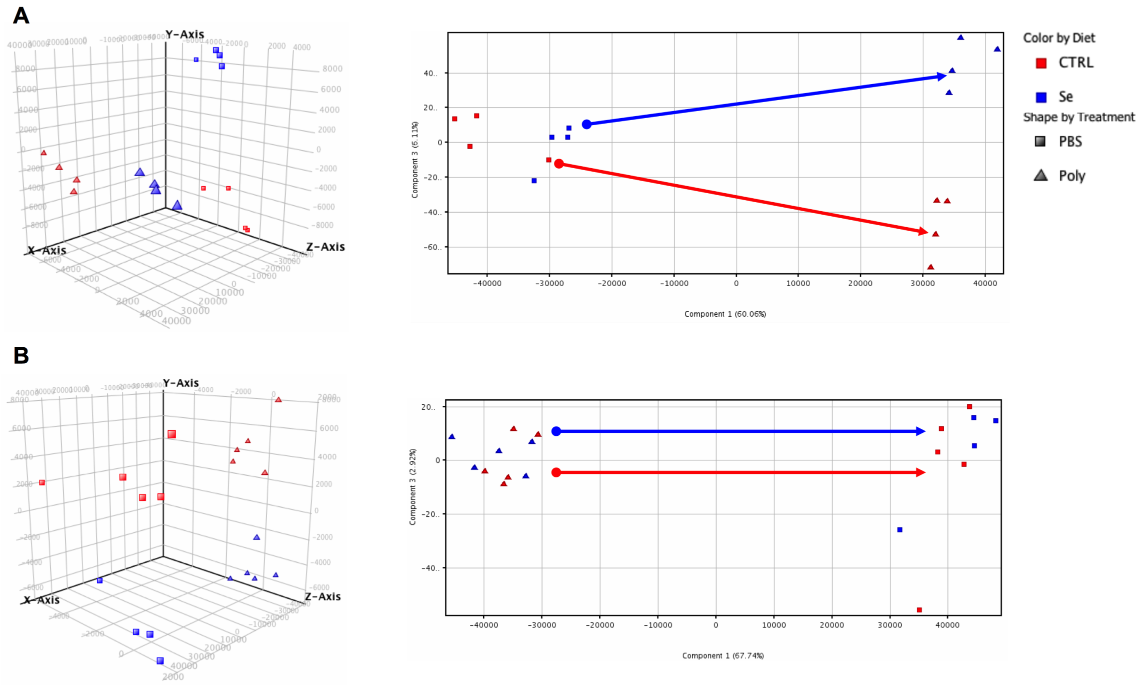

Supplement: Additional file 2: Figure S2. — Principal component analysis of the expression patterns of the four distinct conditions analyzed. Clustering of diets (control diet and Sel-Plex supplementation) and treatments (PBS or poly(I:C)) groups based on the three (left) and two principal components (right), in HK (A) and liver (B). The colours and shapes of the data points indicate diet group and treatment. The x-, y- and z-axes represent PC1, PC2 and PC3, respectively. (PDF 753 kb) [file 12864_2016_2418_MOESM2_ESM.pdf]
