# Supplementary material for: Impact of selenium supplementation on fish antiviral responses: a whole transcriptomic analysis in rainbow trout (Oncorhynchus mykiss) fed supranutritional levels of Sel-Plex®
Source: BMC Genomics. 2016 Feb 16;17:116. doi: 10.1186/s12864-016-2418-7 (PMC4754912; doi:10.1186/s12864-016-2418-7)
Supplement: Additional file 3: Table S1. — Vitamin and mineral composition in the raw material for all diets used in this study. (PDF 61 kb) [file 12864_2016_2418_MOESM3_ESM.pdf]

Additional File 3: Table S1. Vitamin and mineral composition in the raw material for all diets used in this study.

Vitamins and minerals	µg/mg premix
Vitamin A	0
Vitamin D3	0
Vitamin E	58.33
Vitamin B1	3.33
Vitamin B2	6.67
Vitamin B6	3.33
Vitamin B12 (ng/mg)	0.01
Vitamin K3	3.33
Vitamin PP (Niacine)	16.67
Vitamin B3 (Ca D Panthothenic)	13.33
Biotin	0.1
Vitamin C	33.33
Folic acid	3.33
Ethoxyquin	0
Zinc	33.33
Iodine	0.4
Copper	0
Manganese	10
Cobalt	0.33
Selenium mineral	0
