# Supplementary material for: Impact of selenium supplementation on fish antiviral responses: a whole transcriptomic analysis in rainbow trout (Oncorhynchus mykiss) fed supranutritional levels of Sel-Plex®
Source: BMC Genomics. 2016 Feb 16;17:116. doi: 10.1186/s12864-016-2418-7 (PMC4754912; doi:10.1186/s12864-016-2418-7)
Supplement: Additional file 4: Table S2. — Real time qPCR analysis of genes selected for confirmation of microarray data. Candidate transcripts expression in HK (A) and liver (B) RNA isolated from rainbow trout fed a control diet (CTRL) or a diet enriched with 4 mg Se Kg−1 (Sel-Plex), and injected with either PBS or poly(I:C). The expression of gene transcripts was quantified by qPCR and normalized against the geometric mean of three housekeeping genes (ef-1α, drpII, hprt) from the same samples, and then used for statistical analysis. The transcript expression is reported as a fold change, calculated as the average expression level of stimulated samples divided by that of the controls. The results represent the mean ± SEM from five pools of HK or liver RNA extracted from 3 individuals from 3 different tanks. The letters above the columns indicate values that are statistically significant vs the controls (p < 0.05) in the group injected with poly(I:C), with different letters indicating significant differences between the two diet regimes. Asterisks indicate values that are statistically significant vs the control (p < 0.05) in the group fed 4 mg Se Kg−1 and injected with PBS. The primers used are listed in Table 2. (PDF 173 kb) [file 12864_2016_2418_MOESM4_ESM.pdf]

Additional File 4: Table S2. Real time *q*PCR analysis of genes selected for confirmation of microarray data. Candidate transcripts expression in HK (A) and liver (B) RNA isolated from rainbow trout fed a control diet (CTRL) or a diet enriched with 4 mg Se Kg^{-1} (Sel-Plex), and injected with either PBS or poly(I:C). The expression of gene transcripts was quantified by *q*PCR and normalized against the geometric mean of three housekeeping genes (*ef-1α*, *drpII*, *hprt*) from the same samples, and then used for statistical analysis. The transcript expression is reported as a fold change, calculated as the average expression level of stimulated samples divided by that of the controls. The results represent the mean ± SEM from five pools of HK or liver RNA extracted from 3 individuals from 3 different tanks. The letters above the columns indicate values that are statistically significant vs the controls ($p<0.05$) in the group injected with poly(I:C), with different letters indicating significant differences between the two diet regimes. Asterisks indicate values that are statistically significant vs the control ($p<0.05$) in the group fed 4 mg Se Kg^{-1} and injected with PBS. The primers used are listed in Table 2.

A

	IFNγ2		MX2		SOCS3		CATH1		TrxR3b		SelPa	
	Microarray CUST_201_PI429021944	qPCR	Microarray TC149586	qPCR	Microarray IMM 590	qPCR	Microarray IMM 568	qPCR	Microarray CUST_56_PI429042546	qPCR	Microarray CUST_38_PI429042546	qPCR
**Sel-Plex/Poly(I:C)**	19.2	***31.2±4.7*** [a]	28.6	***42.4±12.8*** [a]	14.8	***18.0±4.9***	13.2	***22.8±4.9***	2.6	***2.3±0.2*** [a]	-2.5	***-2.5±0.01*** [a]
**CTRL/Poly(I:C)**	5.9	***12±2.2*** [b]	25.5	***35.2±3.0*** [a]	11.7	***32.6±6.8***	7.6	***11.9±4.9*** [a]	2.2	***3.3±0.1*** [a]	-2.1	***-2.1±0.01*** [a]
**Sel-Plex/PBS**	-1.2	***-1.4±0.1***	-1.0	***0.7±0.1***	1.5	***1.4±0.3***	-1.7	***-1.2±0.1***	1.1	***1.23±0.1***	1.0	***1.2±0.1***

B

	IFN1α		VIG-1		SAA		HAMP		IGFBP-1b		SelPb	
	Microarray TC138564	qPCR	Microarray TC136044	qPCR	Microarray TC171675	qPCR	Microarray CUST_193_PI429021944	qPCR	Microarray CUST_2_PI429021970	qPCR	Microarray CUST_39_PI429042546	qPCR
**Sel-Plex/Poly(I:C)**	2.6	***3.0±0.3*** [a]	31.7	***84.3±6.1*** [a]	82.1	***178.9±44.7*** [a]	13.8	***8.8±1.0*** [a]	-9.1	***-33.3±0.01*** [a]	-2.0	***-2.9±0.03*** [a]
**CTRL/Poly(I:C)**	2.3	***2.6±0.1*** [a]	33.5	***83.4±4.5*** [a]	37.6	***109.6±33.5*** [a]	7.0	***33.2±7.1*** [b]	-9.4	***-25±0.01*** [a]	-2.1	***-2.2±0.06*** [a]
**Sel-Plex/PBS**	1.1	***1.1±0.1***	1.3	***1.4±0.4***	-1.7	***-1.92±0.1***	2.0	***3.3±1.3***	1.6	***-2.1±0.3****	1.1	***1.2±0.1***
