# Supplementary material for: Impact of selenium supplementation on fish antiviral responses: a whole transcriptomic analysis in rainbow trout (Oncorhynchus mykiss) fed supranutritional levels of Sel-Plex®
Source: BMC Genomics. 2016 Feb 16;17:116. doi: 10.1186/s12864-016-2418-7 (PMC4754912; doi:10.1186/s12864-016-2418-7)
Supplement: Additional file 5: Figure S3. — Grouped annotation network (A) and cluster distribution network (B) of the functional terms listed in Fig. 7 visualised using ClueGO. In both layouts, terms are connected depending on the similarity of their associated genes. The degree of connectivity between terms (edges) is calculated using kappa statistics and the network is shown using the Organic layout algorithm supported by Cytoscape. The size of the nodes reflects the statistical significance of the terms, whereas the thickness of the edge reflects the grade of functional connection between terms. The group leading term is the most significant term of the group. In the grouped annotation network, functional terms are grouped by different colours, each identifying the most significant term of the group. In the cluster distribution network, green and red label functional terms are up- and down-regulated genes, respectively. The colour gradient shows the gene proportion of each cluster associated with the term. (PDF 1254 kb) [file 12864_2016_2418_MOESM5_ESM.pdf]

Additional File 5: Figure S3. Grouped annotation network (A) and cluster distribution network (B) of the functional terms listed in Figure 7 visualised using ClueGO. In both layouts, terms are connected depending on the similarity of their associated genes. The degree of connectivity between terms (edges) is calculated using kappa statistics and the network is shown using the Organic layout algorithm supported by Cytoscape. The size of the nodes reflects the statistical significance of the terms, whereas the thickness of the edge reflects the grade of functional connection between terms. The group leading term is the most significant term of the group. In the grouped annotation network, functional terms are grouped by different colours, each identifying the most significant term of the group. In the cluster distribution network, green and red label functional terms are upand down-regulated genes, respectively. The colour gradient shows the gene proportion of each cluster associated with the term.

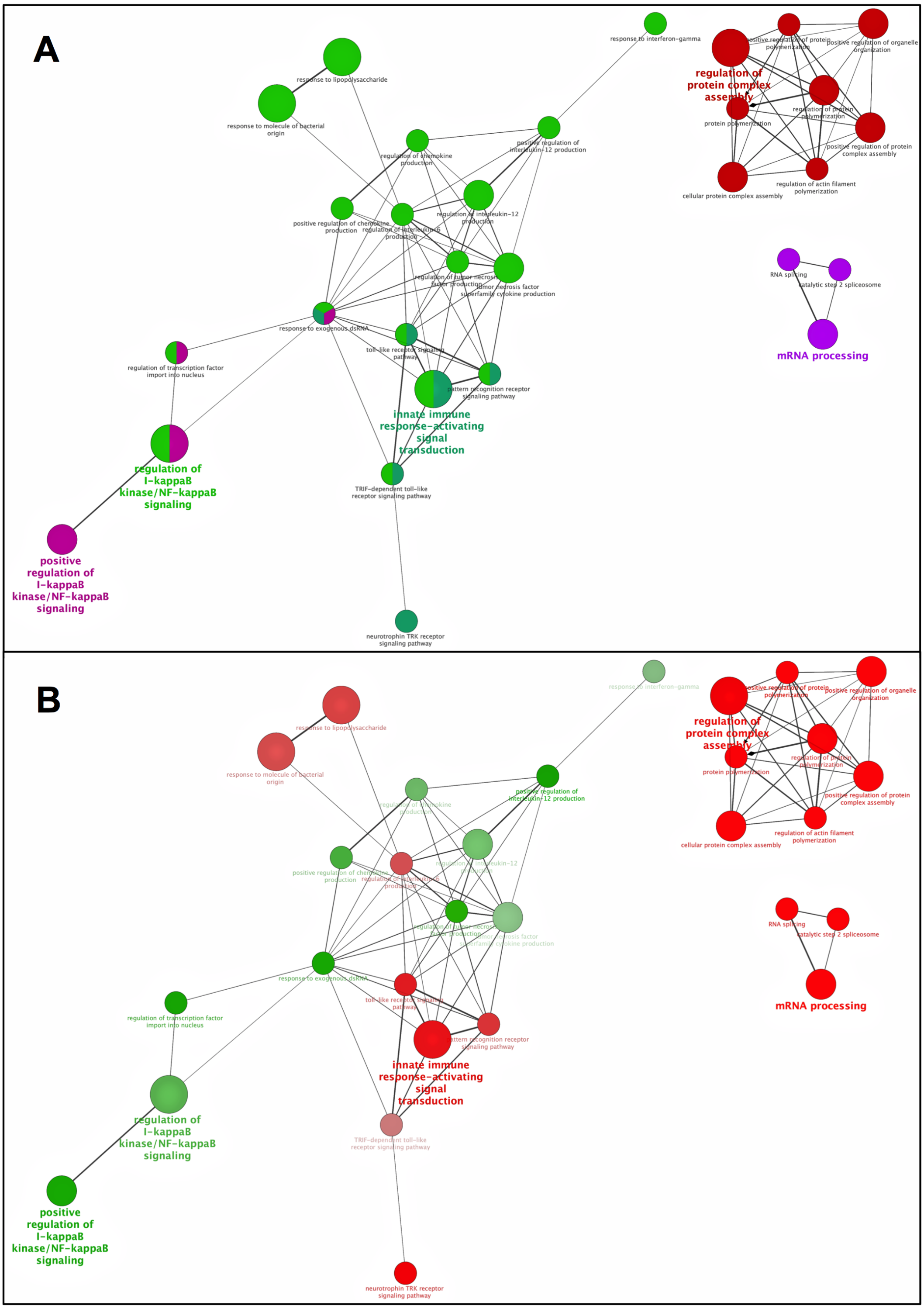

A
response to lipopolysaccharide
response to molecule of bacterial origin
response to interferon-gamma
regulation of chemokine production
positive regulation of interleukin-12 production
positive regulation of chemokine production
regulation of interleukin-6 production
regulation of interleukin-12 production
regulation of tumor necrosis factor production
tumor necrosis factor superfamily cytokine production
response to exogenous dsRNA
regulation of transcription factor import into nucleus
toll-like receptor signaling pathway
pattern recognition receptor signaling pathway
innate immune response-activating signal transduction
regulation of I-kappaB kinase/NF-kappaB signaling
TRIF-dependent toll-like receptor signaling pathway
positive regulation of I-kappaB kinase/NF-kappaB signaling
neurotrophin TRK receptor signaling pathway
regulation of protein complex assembly
positive regulation of protein polymerization
positive regulation of organelle organization
protein polymerization
regulation of protein polymerization
positive regulation of protein complex assembly
cellular protein complex assembly
regulation of actin filament polymerization
RNA splicing
catalytic step 2 spliceosome
mRNA processing
B
response to lipopolysaccharide
response to molecule of bacterial origin
response to interferon-gamma
regulation of chemokine production
positive regulation of interleukin-12 production
positive regulation of chemokine production
regulation of interleukin-6 production
regulation of interleukin-12 production
regulation of tumor necrosis factor production
tumor necrosis factor superfamily cytokine production
response to exogenous dsRNA
regulation of transcription factor import into nucleus
toll-like receptor signaling pathway
pattern recognition receptor signaling pathway
innate immune response-activating signal transduction
regulation of I-kappaB kinase/NF-kappaB signaling
TRIF-dependent toll-like receptor signaling pathway
positive regulation of I-kappaB kinase/NF-kappaB signaling
neurotrophin TRK receptor signaling pathway
regulation of protein complex assembly
positive regulation of protein polymerization
positive regulation of organelle organization
protein polymerization
regulation of protein polymerization
positive regulation of protein complex assembly
cellular protein complex assembly
regulation of actin filament polymerization
RNA splicing
catalytic step 2 spliceosome
mRNA processing
