# Supplementary material for: Impact of selenium supplementation on fish antiviral responses: a whole transcriptomic analysis in rainbow trout (Oncorhynchus mykiss) fed supranutritional levels of Sel-Plex®
Source: BMC Genomics. 2016 Feb 16;17:116. doi: 10.1186/s12864-016-2418-7 (PMC4754912; doi:10.1186/s12864-016-2418-7)
Supplement: Additional file 6: Table S3. — List of transcripts showing the highest change in the HK of fish injected with poly(I:C) and dependent on prior feeding regime. The selection was based on the results of the GO analysis (Fig. 7). All the genes with corresponding microarray feature code1 found involved in a biological process significantly altered by the experimental diet in the HK were selected. If the transcripts were significantly modulated also in fish fed the experimental diet enriched with 4 mg Se Kg−1 by adding Sel-Plex and injected with poly(I:C)3, or in the same tissue of fish fed the control diet and injected with poly(I:C)4 , these values are given. Also the fold change of the expression of the same targets between these last two groups is reported, as given from Genespring software5. All the transcripts shown were significantly modulated at p < 0.05 following the Benjamini-Hochberg correction and had a fold change ≥2. Accession numbers of the cDNA sequence6, their identity7 and the corresponding human orthologue8 determined by BLASTx within the Ensemble database are reported. For each gene the function assigned by ClueGO software is also indicated. SeC represents the groups comparison addressed to analyse the effects of the diet enriched with 4 mg Se Kg−1. CP and SeP instead represent the comparisons addressed to analyse the effect of poly(I:C) stimulation on fish fed either a control diet or the experimental diet respectively. (PDF 145 kb) [file 12864_2016_2418_MOESM6_ESM.pdf]

**Additional File 6: Table S3. List of transcripts showing the highest change in the HK of fish injected with poly(I:C) and dependent on prior feeding regime.** The selection was based on the results of the GO analysis (Figure 7). All the genes with corresponding microarray feature code[1] found involved in a biological process significantly altered by the experimental diet in the HK were selected. If the transcripts were significantly modulated also in fish fed the experimental diet enriched with 4 mg Se $Kg^{-1}$ by adding Sel-Plex and injected with poly(I:C)[3], or in the same tissue of fish fed the control diet and injected with poly(I:C)[4] , these values are given. Also the fold change of the expression of the same targets between these last two groups is reported, as given from Genespring software[5]. All the transcripts shown were significantly modulated at $p<0.05$ following the Benjamini-Hochberg correction and had a fold change ≥2. Accession numbers of the cDNA sequence[6]. Their identity[7] and the corresponding human hortologue[8] determined by BLASTx within the Ensemble database are reported. For each gene the function assigned by ClueGO software is also indicated. SeC represents the groups comparison addressed to analyse the effects of the diet enriched with 4 mg Se $Kg^{-1}$. CP and SeP instead represent the comparisons addressed to analyse the effect of poly(I:C) stimulation on fish fed either a control diet or the experimental diet respectively.

| Trait Identifier[1] | SeC HK[2] | SeP HK[3] | CP HK[4] | SeP[5] / CP | Acc. Number[6] | Identity[7] | HGNC symbol[8] |
|---|---|---|---|---|---|---|---|
| TC163465 | -2.6 | 6.5 | -4.9 | 12.2 | NM_001123548 | Uncharacterized protein LOC100136396 | LALBA |
| TC151657 | 2.1 | -1.1 | -3.8 | 7.8 | BT072640 | Zinc transporter 4 | SLC30A4 |
| TC162033 | -1.1 | 2.6 | -2.2 | 5.2 | BT045008 | Cyclin-G1 | CTR9 |
| TC156274 | 3.3 | -1.8 | -2.3 | 4.4 | XM_003199023 | Protein tyrosine phosphatase receptor type D a | PTPRF |
| TC132729 | -1.5 | 3.8 | -1.4 | 3.6 | BT072208 | BSD domain-containing protein 1 | BSDC1 |
| TC154885 | -1.3 | 4.2 | -1.1 | 3.5 | XM_001344468 | Zinc finger FYVE domain containing 9 | ZFYVE9 |
| TC167981 | 2.2 | 1.3 | -1.2 | 3.4 | BT071997 | Ubiquitin-protein ligase | HECTD1 |
| TC152153 | -1.4 | 4.0 | -1.1 | 3.3 | NM_001146630 | Dysbindin domain-containing protein 1 | DBNDD1 |
| TC157489 | 1.9 | -1.8 | -2.9 | 3.1 | BT072110 | Calcineurin subunit B isoform 1 | PPP3R1 |
| TC137721 | 1.5 | 2.4 | 1.3 | 2.8 | NM_001140813 | E3 ubiquitin-protein ligase | NEURL1B |
| TC162322 | 1.2 | 3.1 | 1.4 | 2.7 | BT047331 | Butyrophilin subfamily 1 member A1 precursor | BTN1A1 |
| TC153681 | -1.3 | 4.9 | 1.3 | 2.7 | not-annotated | not-annotated | GALNT18 |
| TC141145 | 2.6 | -1.2 | -1.3 | 2.7 | BC162106 | Pre-B-cell leukemia transcription factor 4 | PBX1 |
| TC168252 | -1.2 | 1.5 | -2.0 | 2.5 | NM_001140095 | Sialic acid synthase | NANS |
| TC163406 | 2.0 | 1.4 | 1.1 | 2.5 | BT072676 | Myosin 9B | MYO9B |
| TC135281 | 2.1 | 1.1 | -1.1 | 2.5 | BT050147 | Chromosome 7 open reading frame 55 protein | C7orf55 |
| TC142567 | -1.2 | 3.2 | 1.1 | 2.4 | NM_001124710 | Aryl hydrocarbon receptor nuclear translocator | ARNT |
| TC153844 | -1.2 | 2.4 | -1.1 | 2.3 | AB280010 | Runt-related transcription factor 3 | RUNX1 |
| CUST_22_ PI420312184 | -1.6 | 25.9 | 7.4 | 2.2 | NM_001129984 | VHSV-induced protein-8 | CXCL11 |

| Trait Identifier[1] | SeC HK[2] | SeP HK[3] | CP HK[4] | SeP[5] / CP | Acc. Number[6] | Identity[7] | HGNC symbol[8] |
|---|---|---|---|---|---|---|---|
| TC144850 | -1.2 | -8.6 | -5.1 | -2.0 | BT057710 | CN024 protein | FAM177A1 |
| TC142645 | -1.5 | -2.5 | -1.8 | -2.0 | NM_001173956 | $Na^+/K^+$ transporting, beta 4 polypeptide | ATP1B4 |
| TC141261 | -1.1 | -2.7 | -1.5 | -2.0 | BT047980 | Dehydrogenase/reductase SDR family member 13 | DHRS13 |
| TC168836 | -1.7 | -2.3 | -1.9 | -2.1 | AY214171 | Carboxypeptidase H | CPE |
| TC158951 | -1.1 | -4.4 | -2.2 | -2.1 | BT072461 | Unknown large open reading frame | C10orf118 |
| TC171171 | 1.7 | -1.4 | 2.8 | -2.2 | NM_001173805 | C-terminal binding protein 1 | CTBP1 |
| TC134553 | 1.2 | -3.3 | -1.3 | -2.2 | BT056944 | Cellular retinoic acid-binding protein | CRABP2 |
| TC163111 | -1.0 | -2.3 | -1.1 | -2.2 | BT048030 | Eukaryotic translation initiation factor 4E transporter | EIF4ENIF1 |
| TC165621 | -1.2 | -4.2 | -2.2 | -2.3 | BT048535 | Deoxycytidylate deaminase | DCTD |
| TC154495 | 1.2 | -1.2 | 2.5 | -2.3 | NM_001104831 | Tissue-type plasminogen activator | TMPRSS11B |
| TC163157 | 1.0 | -2.2 | 1.1 | -2.3 | BT044862 | Family with sequence similarity 3, member C | FAM3C |
| TC169750 | -1.3 | 1.1 | 2.0 | -2.4 | NM_001165225 | Arachidonate 5-lipoxygenase-activating protein | ALOX5AP |
| TC160815 | -1.1 | -3.3 | -1.5 | -2.5 | not-annotated | not-annotated | CLEC17A |
| TC159034 | -1.3 | -2.3 | -1.1 | -2.7 | NM_001165041 | Programmed cell death 6 | PDCD6 |
| TC143143 | -1.1 | -1.2 | 2.2 | -2.8 | BT058924 | Trifunctional enzyme subunit beta mitochondrial precursor | HADHB |
| TC152317 | 2.7 | 1.2 | 9.6 | -2.8 | NM_001173694 | 6-phosphofructokinase type C | PFKP |
| TC156686 | 1.1 | -3.9 | 1.3 | -4.9 | BC171534 | Uncharacterized protein si:dkey-103i16.1 | PPFIBP1 |
| TC137308 | 2.7 | -1.6 | 9.5 | -5.7 | BT057275 | Mitochondrial import receptor subunit TOM20 homolog | TOMM20 |
| TC132453 | -1.3 | -1.8 | 2.5 | -5.9 | NM_001124691 | Tubulin alpha chain testis-specific (LOC100136736) | TUBA3D |
