# Supplementary material for: Impact of selenium supplementation on fish antiviral responses: a whole transcriptomic analysis in rainbow trout (Oncorhynchus mykiss) fed supranutritional levels of Sel-Plex®
Source: BMC Genomics. 2016 Feb 16;17:116. doi: 10.1186/s12864-016-2418-7 (PMC4754912; doi:10.1186/s12864-016-2418-7)
Supplement: Additional file 7: Table S5. — List of selenoprotein transcripts significantly modulated. All the selenoprotein transcripts modulated in the HK and liver of fish fed the experimental diet enriched with 2 g Kg−1 Sel-Plex and injected with PBS, of fish injected with poly(I:C) and fed the experimental diet enriched with 2 g Kg−1 Sel-Plex and the control diet respectively, are given. All the transcripts shown were significantly modulated (p < 0.05) following the Benjamini–Hochberg correction and had a fold change ≥2. Accession numbers of the cDNA sequences6, their identity7 and the corresponding human orthologue8 determined by BLASTx and BLASTn are reported. SeC represents the groups comparison addressed to analyse the effects of the 2 g Kg−1 Sel-Plex diet. CP and SeP instead represent the comparisons addressed to analyse the effect of poly(I:C) stimulation on fish fed either a control diet or the 2 g Kg−1 Sel-Plex diet respectively. (PDF 111 kb) [file 12864_2016_2418_MOESM7_ESM.pdf]

Additional File 7: Table S5. List of selenoprotein transcripts significantly modulated. All the selenoprotein transcripts modulated in the HK and liver of fish fed the experimental diet enriched with 4 g Kg^{-1} Sel-Plex and injected with PBS, of fish injected with poly(I:C) and fed the experimental diet enriched with 4 g Kg^{-1} Sel-Plex and the control diet respectively, are given. All the transcripts shown were significantly modulated ($p<0.05$) following the Benjamini–Hochberg correction and had a fold change ≥2. Accession numbers of the cDNA sequences[6], their identity[7] and the corresponding human orthologue[8] determined by BLASTx and BLASTn are reported. SeC represents the groups comparison addressed to analyse the effects of the 4 g Kg^{-1} Sel-Plex diet. CP and SeP instead represent the comparisons addressed to analyse the effect of poly(I:C) stimulation on fish fed either a control diet or the 4 g Kg^{-1} Sel-Plex respectively.

Trait[1] Identifier	SeC[2]	CP[3]	CP[4]	Acc. Number[5]	Identity[6]	HGNC symbol[7]
**HK**						
CUSR_21_PI	1.0	-4.7	-4.3	not-annotated	Type I iodothyronine deiodinase	DIO1
TC133469	-1.1	2.2	1.9	NM_0011245	Glutathione peroxidase type 2	GPX2
CUST_38_PI	1.0	-2.1	-2.0	HF969249	Selenoprotein Pa	SEPP1
TC164235	1.1	2.7	2.3	BT072601	Thioredoxine reductase 1	TXNRD1
**Liver**						
TC134139	-1.3	-5.6	-4.7	AF207900	Type II iodothyronine deiodinase	DIO2
TC132596	1.4	-2.2	-1.5	NM_0011245	Glutathione peroxidase type 2	GPX2
CUST_39_PI	1.2	-2.1	-2.0	HF969250	Selenoprotein Pb	SEPP1
TC154252	1.2	-2.1	-1.7	BT045754	tRNA slenocysteine-associated protein 1	TRNAU1AP
